# Electronic Health Record–Based Absolute Risk Prediction Model for Esophageal Cancer in the Chinese Population: Model Development and External Validation

**DOI:** 10.2196/43725

**Published:** 2023-03-15

**Authors:** Yuting Han, Xia Zhu, Yizhen Hu, Canqing Yu, Yu Guo, Dong Hang, Yuanjie Pang, Pei Pei, Hongxia Ma, Dianjianyi Sun, Ling Yang, Yiping Chen, Huaidong Du, Min Yu, Junshi Chen, Zhengming Chen, Dezheng Huo, Guangfu Jin, Jun Lv, Zhibin Hu, Hongbing Shen, Liming Li

**Affiliations:** 1 Department of Epidemiology and Biostatistics School of Public Health Peking University Beijing China; 2 Department of Epidemiology, Center for Global Health, School of Public Health, Nanjing Medical University Nanjing China; 3 Jiangsu Key Lab of Cancer Biomarkers, Prevention and Treatment, Collaborative Innovation Center for Cancer Medicine, China International Cooperation Center for Environment and Human Health, Nanjing Medical University Nanjing China; 4 Peking University Center for Public Health and Epidemic Preparedness and Response Beijing China; 5 Chinese Academy of Medical Sciences Beijing China; 6 Medical Research Council Population Health Research Unit, University of Oxford Oxford United Kingdom; 7 Clinical Trial Service Unit and Epidemiological Studies Unit, Nuffield Department of Population Health, University of Oxford Oxford United Kingdom; 8 Zhejiang Center for Disease Control and Prevention Hangzhou China; 9 China National Center for Food Safety Risk Assessment Beijing China; 10 Department of Public Health Sciences, The University of Chicago Chicago, IL United States

**Keywords:** esophageal cancer, prediction model, absolute risk, China, prospective cohort, screening, primary prevention, development, external validation, electronic health record

## Abstract

**Background:**

China has the largest burden of esophageal cancer (EC). Prediction models can be used to identify high-risk individuals for intensive lifestyle interventions and endoscopy screening. However, the current prediction models are limited by small sample size and a lack of external validation, and none of them can be embedded into the booming electronic health records (EHRs) in China.

**Objective:**

This study aims to develop and validate absolute risk prediction models for EC in the Chinese population. In particular, we assessed whether models that contain only EHR-available predictors performed well.

**Methods:**

A prospective cohort recruiting 510,145 participants free of cancer from both high EC-risk and low EC-risk areas in China was used to develop EC models. Another prospective cohort of 18,441 participants was used for validation. A flexible parametric model was used to develop a 10-year absolute risk model by considering the competing risks (full model). The full model was then abbreviated by keeping only EHR-available predictors. We internally and externally validated the models by using the area under the receiver operating characteristic curve (AUC) and calibration plots and compared them based on classification measures.

**Results:**

During a median of 11.1 years of follow-up, we observed 2550 EC incident cases. The models consisted of age, sex, regional EC-risk level (high-risk areas: 2 study regions; low-risk areas: 8 regions), education, family history of cancer (simple model), smoking, alcohol use, BMI (intermediate model), physical activity, hot tea consumption, and fresh fruit consumption (full model). The performance was only slightly compromised after the abbreviation. The simple and intermediate models showed good calibration and excellent discriminating ability with AUCs (95% CIs) of 0.822 (0.783-0.861) and 0.830 (0.792-0.867) in the external validation and 0.871 (0.858-0.884) and 0.879 (0.867-0.892) in the internal validation, respectively.

**Conclusions:**

Three nested 10-year EC absolute risk prediction models for Chinese adults aged 30-79 years were developed and validated, which may be particularly useful for populations in low EC-risk areas. Even the simple model with only 5 predictors available from EHRs had excellent discrimination and good calibration, indicating its potential for broader use in tailored EC prevention. The simple and intermediate models have the potential to be widely used for both primary and secondary prevention of EC.

## Introduction

China has the largest burden of esophageal cancer (EC), accounting for around half of the global incident cases and deaths in 2018 [1,2]. The prevalence, disability-adjusted life years, and direct medical expenditures are projected to continue to increase [3]. Upper endoscopy has been widely performed for screening and diagnosing EC, but the cost and potential harm of invasive procedures as well as the need for expertise and endoscopy skills training preclude a population-wide application, which may partially explain the poor prognosis of EC. Thus, identifying a high-risk population for endoscopy through prediction models would be more feasible and effective.

In China, 4 diagnostic models (ie, estimating the probability of prevalent EC) have been developed to act as a prescreening tool for endoscopy, with an area under the receiver operating characteristic curve (AUC) ranging from 0.681 to 0.843 [4-7]. However, these models were all developed from populations in high-risk rural areas and may not apply to low-risk rural and urban areas, where a large proportion of cases countrywide occurs [8]. Only 1 model was externally validated [5]. Besides diagnostic models, a few prognostic models (ie, predicting the absolute risk of EC in 5 or more years) have recently been developed from European cohorts [9-12]. These models can be used not only for early detection but also for primary prevention such as intensive lifestyle interventions. However, such models may not perform well for the Chinese population since the risk factor profile is different and the predominant subtype of EC is esophageal squamous cell carcinoma (ESCC) among the Chinese, while esophageal adenocarcinoma is the predominant subtype in the western population [13]. To the best of our knowledge, there is only 1 prognostic model in China, which was developed in a high-risk rural area [14]; this model was based on a case-control design, which was prone to selection bias and recall bias. Moreover, the limited EC cases (n=244) and the lack of external validation could induce overfitting and encroach generalizability.

The national Basic Public Health Service program in China requires establishing health records for all residents [15]. The efficiency and extensive use of population risk stratification for tailored prevention can be greatly improved by embedding prediction models within the electronic health record (EHR) system, that is, by directly estimating the risk of EC and identifying high-risk individuals for EC based on information from EHRs. However, some predictors in the existing models (eg, food temperature, eating speed) are not available in EHRs and need to be additionally collected even in high-risk areas of EC.

To address the above limitations, we used a large prospective cohort of 0.5 million people from both high EC-risk and low EC-risk areas of China for model development and another prospective cohort for external validation. We first constructed a 10-year absolute risk prediction model for EC with the inclusion of established and probable EC risk factors to maximize model performance. Then, we simplified the model by keeping predictors that are available in the Basic Public Health Service health records. We assessed whether the simple but potentially widely applicable model showed acceptable performance in both cohorts.

## Methods

### Data for Model Development

Data from the China Kadoorie Biobank (CKB), a large-scale nationwide prospective cohort of 512,725 participants aged 30-79 years, were used for model development. The baseline survey was performed between 2004 and 2008 in 10 geographically defined regions (5 urban and 5 rural). The details of the study design and survey methods have been reported previously [16]. Incident cases of EC and all-cause mortality were identified through linkage with the mortality and disease registries and national health insurance claims database, supplemented with local residential records and annual active confirmation. The International Classification of Diseases, 10th revision was used to code all EC (C15) by trained staff who were blinded to the baseline information. The adjudication of the incident cancer cases is ongoing, with medical records of 1283 EC cases having been retrieved, in which 1246 (97.1%) were confirmed as EC and 830 had pathological diagnoses. After excluding 41 cases with subtype reported as unknown, 92.7% (731/789) of the cases were classified as ESCC.

### Data for External Validation

An independent prospective cohort from Changzhou of the Jiangsu province, a low EC-risk rural area in China, was used for external validation. In brief, 20,803 participants aged 30 years and older were recruited from 23 villages in 2004-2005. Incident EC cases and all-cause mortality were identified through active follow-up in 2008-2009, 2012-2013, and 2018-2019, and through linkage with the disease and mortality registries. Trained staff who were blinded to baseline information further confirmed suspected cases of nonfatal cancer by reviewing local medical records or visiting village doctors.

### Ethics Approval

The study protocol for CKB was approved by the ethics review committee of the Chinese Center for Disease Control and Prevention (Beijing, China: 005/2004) and the Oxford Tropical Research Ethics Committee, University of Oxford (UK: 025–04). The Changzhou cohort was approved by the ethical review committee of the Nanjing Medical University (Nanjing, China), and written informed consent was collected from all the participants.

### Predictor Variables

At baseline, all participants in the CKB and Changzhou cohort completed a questionnaire and had physical measurements taken. Candidate predictors were identified based on established risk factors for EC and factors that have been included in previous EC prediction models [17,18]. Candidate predictors included age, sex, smoking, alcohol use, education, household income, marital status, family history of EC, BMI, waist circumference, physical activity, hot food consumption, and consumption of fresh vegetables, fresh fruit, red meat, and preserved vegetables. To model the large geographic disparity in EC incidence in China, we created a variable to denote living in a high-risk or low-risk area. Of the 10 study regions of CKB, we assigned Hui county in Henan province and Pengzhou in Sichuan province to high-risk areas, according to the most recent guideline for EC in China [19,20]. The criteria for defining high-risk areas are described in Multimedia Appendix 1 [19,21-26]. The details of baseline prevalence and incidence of EC by study region are shown in Multimedia Appendix 2. Because data on the family history of EC and hot food consumption were not recorded at baseline in the CKB, we used family history of cancer and hot tea consumption as surrogates for the above-established risk factors. The details of the assessment of predictors are shown in Multimedia Appendix 1.

### Statistical Methods

In the CKB cohort, participants who were previously diagnosed with cancer (n=2578) or had missing data on BMI (n=2) were excluded, leaving 510,145 participants for development. In the Changzhou cohort, participants who were previously diagnosed with cancer (n=239), out of the age range of 30-79 years (n=1902), had a recorded implausible censoring date for loss to follow-up (n=5), or had missing data on candidate predictors (n=216) were excluded, leaving 18,441 participants for external validation. Participants were considered at risk from enrollment to the first date of diagnosis of EC, death, loss to follow-up, or end of follow-up (CKB: December 31, 2017; Changzhou cohort: January 31, 2019).

### Model Development

Based on the whole CKB data set, we separately fitted a model for EC and a model for all-cause mortality. For the EC model, a flexible parametric model on the cumulative hazard scale was used to estimate the baseline hazards and hazard ratios of the predictors for EC, with age as the time scale [27]. Age was modeled using restricted cubic splines with boundary knots at 30 and 90 and internal knots at 60 and 70. The established risk factors of EC (age, sex, smoking, and alcohol use) and regional risk level (high-risk/low-risk areas) entered the model directly. Two strategies were employed for the selection of the other predictors. First, other candidate predictors were included in the full model and kept if *P*<.05. Second, the predictor selection was repeated using stepwise backward elimination. Two strategies selected the same set of predictors. The variable grouping was determined using the Bayesian information criteria. All 2-way interactions were tested, but none of those significantly improved model performance. Further, we simplified the full model by keeping only predictors available in the health records. As age is the most important predictor, we also constructed an age-only model for comparison. Therefore, 4 models were constructed, with predictors included in the model: (1) age-only: age; (2) simple model: age, sex, regional risk level, education, and family history of cancer, which are available for all residents in the health records; (3) intermediate model: simple model plus smoking, alcohol use, and BMI, which are additionally available for residents aged 65 years and older, and diabetic or hypertensive patients in the health records; (4) full model: intermediate model plus physical activity, hot tea consumption, and fresh fruit consumption, which go beyond the available health records but have the potential to improve the risk prediction. We then used the same settings of the flexible parametric model to model the hazards of all-cause mortality, with sex, residence area (urban/rural), and regional risk level in the model. We used cause-specific hazard models to account for the competing risks. Briefly, the 10-year absolute risk (AR) of EC for a participant who is age a is calculated as



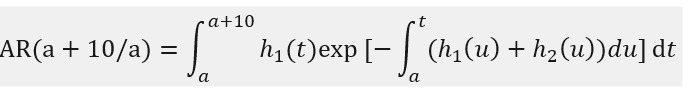



### Model Validation

The methods for model validation are detailed in Multimedia Appendix 1. In brief, we externally validated the age-only, simple, and intermediate models, but not the full model, because data on physical activity in metabolic equivalent of task-hours and hot tea consumption were unavailable in the Changzhou cohort. We also conducted an internal validation in the CKB by using data splitting and 500-sample bootstrapping. Cancer-free participants whose retention in the cohorts was less than 10 years were included to test calibration but were excluded from other validation measures, since it was unknown whether they could have experienced an EC if they had been followed up to 10 years.

Discrimination was quantified by calculating the AUC. Calibration was assessed by plotting the observed risk obtained using Kaplan-Meier analyses against the predicted risk by decile. Because of the large geographical variation in the incidence of EC in China, we recalibrated the models by using the method proposed by the World Health Organization Cardiovascular Disease Risk Chart Working Group with a slight modification [21]. Further, continuous Net Reclassification Improvement and Integrated Discrimination Improvement were used to evaluate the added predictive ability of additional predictors [28,29]. In the internal validation using data splitting, calibration and discrimination were also assessed in subgroups defined by regional risk level, residence area, sex, age group, and special population aged 65 years and older or with diabetes or hypertension who are of particular concern to the Basic Public Health Service. To offer a reference for primary care practices, we estimated a range of performance indices corresponding to a series of cutoffs.

Several sensitivity analyses were conducted. First, we separately developed 2 models for high EC-risk (high-risk model) and low EC-risk (low-risk model) areas by using the same strategy as the primary analyses and assessed their discrimination and calibration in the corresponding areas. Second, we restricted EC cases to (1) pathologically confirmed cases, (2) cases that were pathologically confirmed as ESCC, (3) cases that were pathologically confirmed but not as ESCC, and (4) cases that were pathologically confirmed but not as ESCC (scenario 3) or that were not pathologically confirmed. In the above 4 scenarios, we excluded EC cases that did not meet the corresponding criteria and examined the discriminating ability of the models (Multimedia Appendix 3). Third, since some asymptomatic EC cases might be undiagnosed, we excluded the EC cases documented in the first year of follow-up and used the same strategy to develop and validate the models.

## Results

The mean age of the 510,145 participants in the CKB and 18,441 participants in the Changzhou cohort was 52.0 (SD 10.7) years and 51.2 (SD 12.1) years, respectively. The details of the baseline characteristics of the predictors are described in Table 1. During a median of 11.1 (IQR 10.2-12.1) years of follow-up of the CKB, we identified 2550 EC cases, with an incidence (per 100,000 person-years) of 46.2. High EC-risk areas had a significantly higher incidence than low EC-risk areas (132.2 vs 20.2, respectively). In the Changzhou cohort, 114 EC cases were identified during a median follow-up of 13.6 (IQR 13.5-14.4) years, with an incidence of 47.1.

Table 2 and Multimedia Appendix 4 list the hazard ratios and 95% CIs for predictors of EC and all-cause mortality in the CKB. Male, living in high-risk areas, less educated, having a family history of cancer, smoking, alcohol use, underweight, less physical activity, preferring burning hot tea, and rare intake of fresh fruits were associated with a greater risk of EC.

**Figure 1:**
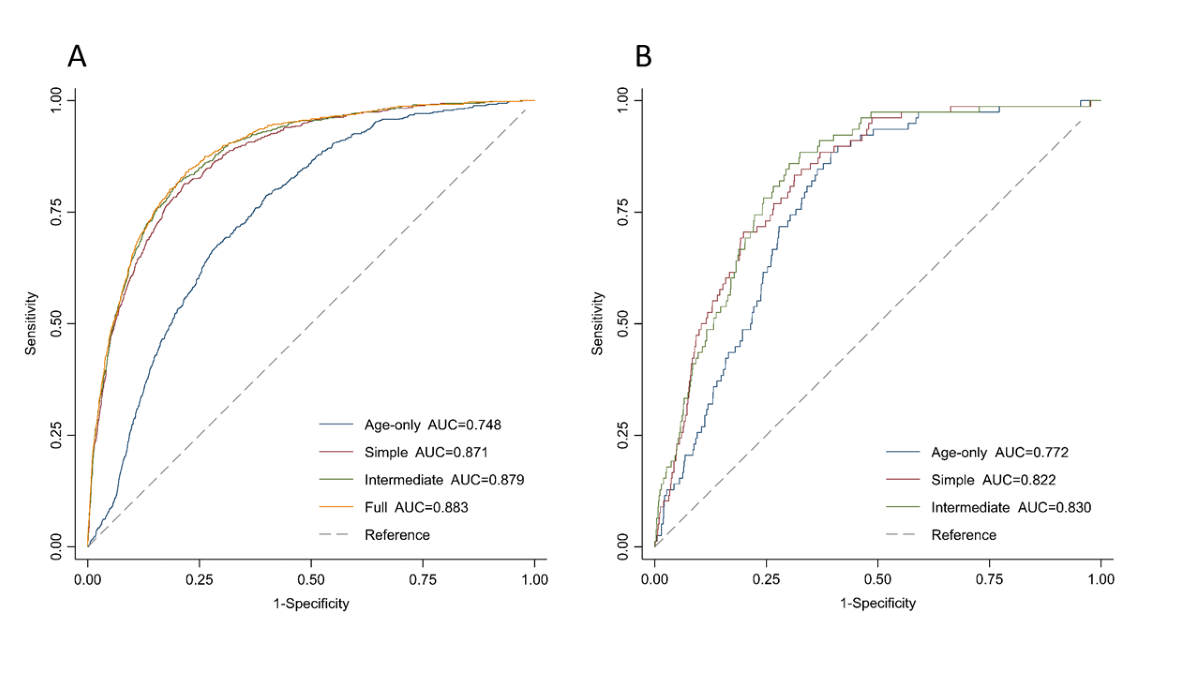
Receiver operating characteristic curves and corresponding areas under the receiver operating characteristic curve for the esophageal cancer prediction models. (A) Internal validation in the China Kadoorie Biobank using data splitting. (B) External validation in the Changzhou cohort. The models included age (age-only model), sex, regional risk level, education, family history of cancer (simple model), smoking, alcohol use, BMI (intermediate model), physical activity, hot tea consumption, and fresh fruit consumption (full model). AUC: area under the receiver operating characteristic curve.

**Figure 2:**
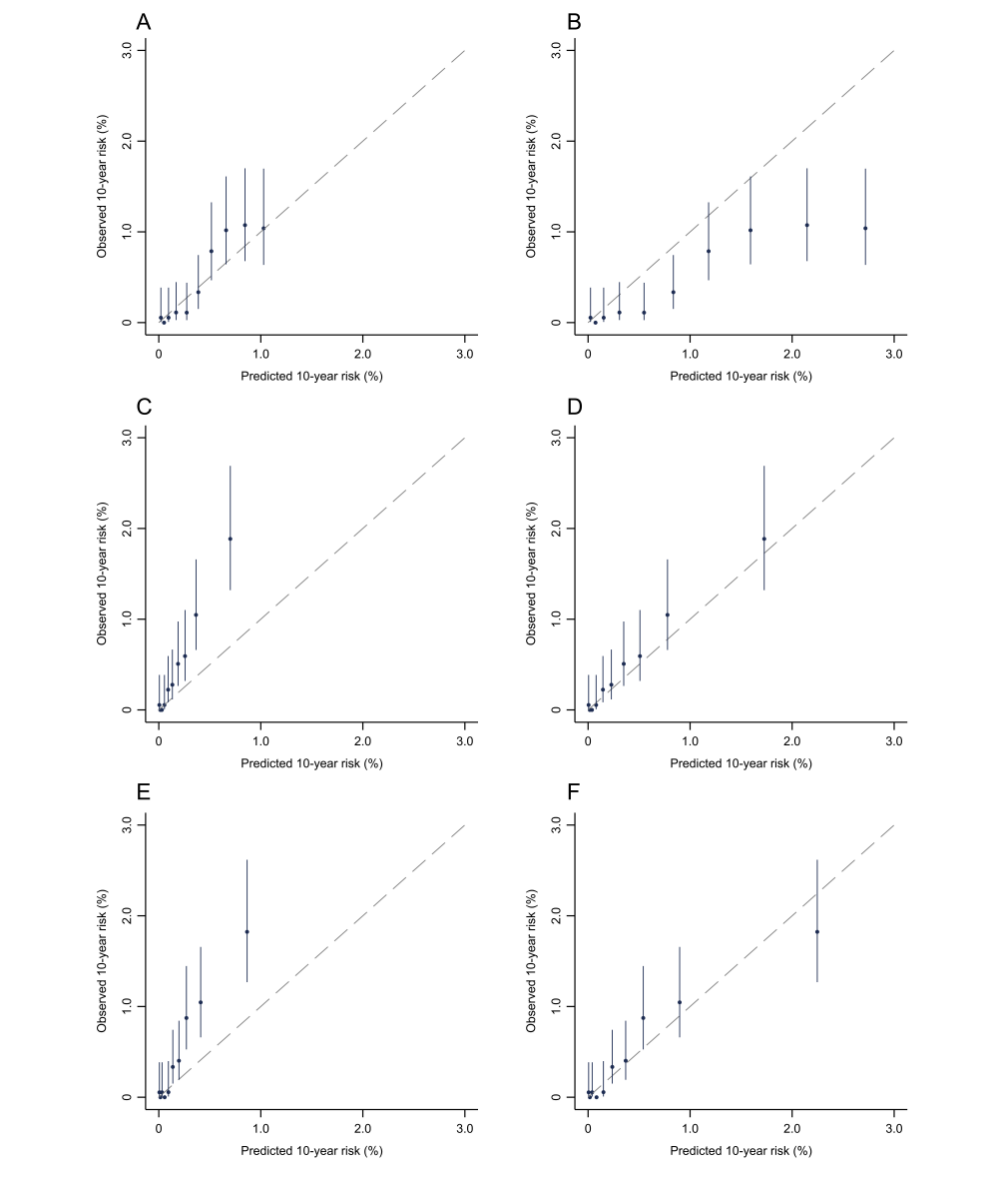
Calibration plot of the esophageal cancer prediction models in the Changzhou cohort. Calibration of the original (A) age-only, (C) simple, and (E) intermediate models. Calibration of the recalibrated (B) age-only, (D) simple, and (F) intermediate models. The observed 10-year risk was estimated by Kaplan-Meier analysis and plotted against model-predicted risk by decile. Models were recalibrated using the method proposed by the World Health Organization Cardiovascular Disease Risk Chart Working Group with a slight modification. For details, see Multimedia Appendix 1.

**Figure 3:**
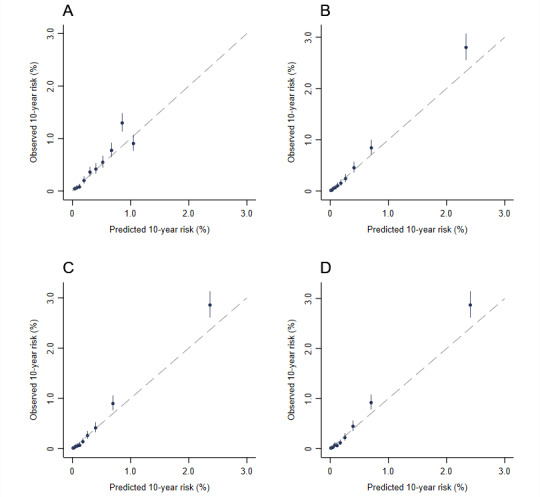
Calibration plot of the esophageal cancer prediction models in the China Kadoorie Biobank by using data splitting. (A) Age-only model. (B) Simple model. (C) Intermediate model. (D) Full model. Models were fitted to a random two-thirds of the China Kadoorie Biobank data and evaluated on the remaining one-third. The calibration plots in the validation set were plotted. The observed 10-year risk was estimated by Kaplan-Meier analyses and plotted against model-predicted risk by decile.

In the external validation, the simple and intermediate models exhibited similar and excellent discriminating ability with AUCs (95% CIs) of 0.822 (0.783-0.861) and 0.830 (0.792-0.867), respectively (Figure 1). In the internal validation, the AUCs (95% CIs) of the simple, intermediate, and full models were 0.871 (0.858-0.884), 0.879 (0.867-0.892), and 0.883 (0.871-0.895), respectively (Figure 1). Although there were only limited increases in the AUCs with more predictors included in the models, continuous Net Reclassification Improvement and Integrated Discrimination Improvement indicated improved accuracy of the predicted risks for both cases and those that were not cases (Multimedia Appendix 5). In the less biased internal validation method of bootstrapping, the above results were not greatly altered (Multimedia Appendix 6). The original simple and intermediate models significantly underestimated the risk of EC in the Changzhou cohort. The recalibration parameters, *b* and *k*, were 1.22 and 1.97, respectively. Age-specific observed risks of EC used to calculate *b* and *k* are shown in Multimedia Appendix 7. After recalibration, the calibration plot showed excellent agreement between the observed and predicted risks for the simple and intermediate models (Figure 2). In the internal validation, the predicted risk of the simple, intermediate, and full models agreed well with the observed risk by a tenth of the predicted risk, except for the top 2 deciles where slight underestimations seemed to have occurred (Figure 3).

The density of the predicted risks of models in cases was greater than that in those that were not cases (Multimedia Appendix 8 and Multimedia Appendix 9). The performance of the models across a series of cutoffs is presented in Multimedia Appendix 10. Compared with their counterparts, the models discriminated better in low-risk areas, rural areas, women, or middle-aged adults younger than 65 years without diabetes and hypertension in the internal validation (Multimedia Appendix 11). The predicted risks agreed well with the observed risks in all subgroups.

In the sensitivity analysis, we separately developed 2 models for high-risk and low-risk areas. The included predictors and the hazard ratios (95% CIs) are listed in Multimedia Appendix 12. When these 2 models were applied in their corresponding validation set, the model for low-risk areas performed better than the models in the primary analyses (Multimedia Appendix 5 and Multimedia Appendix 13). When we took the availability and results of pathology reports into consideration, models had excellent discriminating ability in all scenarios (Multimedia Appendix 3 and Multimedia Appendix 14). Excluding EC cases occurring in the first year of follow-up did not alter the performance of the models (Multimedia Appendix 15).

**Table 1:** Baseline characteristics of the participants by disease status in the China Kadoorie Biobank and Changzhou cohort.

		China Kadoorie Biobank				Changzhou cohort		
		EC^a^ case (n=2550)	Not an EC case (n=507,595)	Total (N=510,145)	EC case (n=114)		Not an EC case (n=18,327)	Total (N=18,441)
Age (years), mean (SD)		60.7 (8.7)	52.0 (10.7)	52.0 (10.7)	60.9 (9.1)		51.1 (12.1)	51.2 (12.1)
Male, n (%)		1757 (68.9)	207,477 (40.9)	209,234 (41)	77 (67.5)		7611 (41.5)	7688 (41.7)
Urban, n (%)		468 (18.4)	224,300 (44.2)	224,768 (44.1)	0 (0)		0 (0)	0 (0)
High-risk area^b^, n (%)		1692 (66.4)	116,715 (23)	118,407 (23.2)	0 (0)		0 (0)	0 (0)
Family history of cancer, n (%)		720 (28.2)	84,948 (16.7)	85,668 (16.8)	26 (22.8)		3354 (18.3)	3380 (18.3)
High level of physical activity^c^, n (%)		565 (22.2)	126,739 (25)	127,304 (25)	—^d^		—	—
**Highest education, n (%)**								
	Illiterate or primary school	1917 (75.2)	257,088 (50.6)	259,005 (50.8)	70 (61.4)		8372 (45.7)	8442 (45.8)
	Middle or high school	598 (23.5)	220,780 (43.5)	221,378 (43.4)	43 (37.7)		9841 (53.7)	9884 (53.6)
	College or university	35 (1.4)	29,727 (5.9)	29,762 (5.8)	1 (0.9)		114 (0.6)	115 (0.6)
**Current smoking (cigarettes or equivalent per day), n (%)**								
	<30	979 (38.4)	114,815 (22.6)	115,794 (22.7)	49 (43)		4230 (23.1)	4279 (23.2)
	≥30	235 (9.2)	19,155 (3.8)	19,390 (3.8)	8 (7)		713 (3.9)	721 (3.9)
**Daily alcohol use (grams of pure alcohol per day), n (%)**								
	<30	70 (2.7)	11,503 (2.3)	11,573 (2.3)	9 (7.9)		1123 (6.1)	1132 (6.1)
	30-59	161 (6.3)	14,884 (2.9)	15,045 (2.9)	9 (7.9)		1049 (5.7)	1058 (5.7)
	≥60	386 (15.1)	19,085 (3.8)	19,471 (3.8)	31 (29)		1956 (10.7)	1989 (10.8)
**BMI (kg/m^2^), n (%)**								
	<18.5	175 (6.9)	21,965 (4.3)	22,140 (4.3)	4 (3.5)		984 (5.4)	988 (5.4)
	18.5-23.9	1482 (58.1)	263,169 (51.8)	264,651 (51.9)	76 (66.7)		9967 (54.4)	10,043 (54.4)
	≥24.0	893 (35)	222,461 (43.8)	223,354 (43.8)	34 (29.8)		7376 (40.3)	7410 (40.2)
**Tea temperature preference, n (%)**								
	Not daily drinker/warm tea drinker	2090 (82)	426,628 (84)	428,718 (84)	—		—	—
	Hot tea	311 (12.2)	59,425 (11.7)	59,736 (11.7)	—		—	—
	Burning hot tea	149 (5.8)	21,542 (4.2)	21,691 (4.3)	—		—	—
**Fresh fruit consumption^e^, n (%)**								
	Daily	165 (6.5)	95,715 (18.9)	95,880 (18.8)	4 (3.5)		813 (4.4)	817 (4.4)
	Weekly	658 (25.8)	207,716 (40.9)	208,374 (40.8)	98 (86)		15,921 (86.9)	16,019 (86.9)
	Less than weekly	1727 (67.7)	204,164 (40.2)	205,891 (40.4)	12 (10.5)		1591 (8.7)	1603 (8.7)

^a^EC: esophageal cancer.

^b^High-risk area denotes Hui county in Henan province and Pengzhou in Sichuan province in our study.

^c^High-level physical activity was defined as age-specific and sex-specific upper quarter of total physical activity level measured by metabolic equivalent of task-hours per day.

^d^Not available.

^e^Data on the fresh fruit consumption of 2 participants in the Changzhou cohort were missing.

**Table 2:** Hazard ratios (95% CIs) for the predictor variables of esophageal cancer in the China Kadoorie Biobank.

			Cases (n)		Cases/person years (1/100,000)		Age-only model, HR^a^ (95% CI)		Simple model, HR (95% CI)		Intermediate model, HR (95% CI)		Full model, HR (95% CI)
**Spline basis of age (knots: 30, 60, 70, 90)**													
	First	N/A^b^		N/A		3.56 (3.29-3.86)		3.28 (3.03-3.56)		3.30 (3.04-3.59)		3.28 (3.02-3.56)	
	Second	N/A		N/A		1.17 (1.11-1.23)		1.15 (1.09-1.21)		1.14 (1.09-1.20)		1.14 (1.09-1.20)	
	Third	N/A		N/A		1.02 (1.00-1.04)		1.00 (0.98-1.02)		1.00 (0.98-1.02)		1.00 (0.98-1.02)	
**Sex**													
	Male	1757		79.12		N/A		Reference		Reference		Reference	
	Female	793		24.02		N/A		0.31 (0.28-0.34)		0.40 (0.36-0.44)		0.42 (0.37-0.46)	
**High-risk area^c^**													
	No	858		20.22		N/A		Reference		Reference		Reference	
	Yes	1692		132.22		N/A		6.31 (5.81-6.86)		6.07 (5.58-6.60)		5.61 (5.14-6.13)	
**Highest education**													
	Illiterate or primary school	1917		69.06		N/A		Reference		Reference		Reference	
	Middle or high school	598		24.68		N/A		0.60 (0.55-0.66)		0.65 (0.59-0.72)		0.68 (0.62-0.76)	
	College or university	35		10.82		N/A		0.32 (0.23-0.44)		0.37 (0.26-0.52)		0.45 (0.32-0.63)	
**Family history of cancer**													
	No	1830		39.88		N/A		Reference		Reference		Reference	
	Yes	720		77.14		N/A		1.71 (1.57-1.86)		1.78 (1.63-1.94)		1.74 (1.59-1.89)	
**Current smoking**													
	No	1336		32.72		N/A		N/A		Reference		Reference	
**Cigarettes or equivalent per day among smokers**													
	<30	979		79.48		N/A		N/A		1.15 (1.05-1.27)		1.12 (1.02-1.24)	
	≥30	235		112.95		N/A		N/A		1.25 (1.07-1.47)		1.24 (1.06-1.45)	
**Daily alcohol use**													
	No	1933		38.43		N/A		N/A		Reference		Reference	
**Grams of pure alcohol per day among alcohol consumers**													
	<30	70		56.84		N/A		N/A		0.97 (0.77-1.24)		1.03 (0.81-1.30)	
	30-59	161		100.03		N/A		N/A		1.39 (1.18-1.64)		1.43 (1.21-1.69)	
	≥60	386		185.48		N/A		N/A		2.01 (1.78-2.26)		2.06 (1.82-2.32)	
**BMI (kg/m^2^)**													
	<18.5	175		77.77		N/A		N/A		Reference		Reference	
	18.5-23.9	1482		51.70		N/A		N/A		0.73 (0.62-0.85)		0.76 (0.65-0.88)	
	≥24.0	893		36.73		N/A		N/A		0.61 (0.52-0.72)		0.64 (0.54-0.76)	
**Physical activity**													
	Low	1985		48.10		N/A		N/A		N/A		Reference	
	High^d^	565		40.49		N/A		N/A		N/A		0.80 (0.73-0.88)	
**Tea temperature preference**													
	Not a daily drinker or warm tea drinker	2090		44.96		N/A		N/A		N/A		Reference	
	Hot tea	311		48.27		N/A		N/A		N/A		1.03 (0.91-1.17)	
	Burning hot tea	149		64.89		N/A		N/A		N/A		1.49 (1.25-1.77)	
**Fresh fruit consumption**													
	Daily	165		15.74		N/A		N/A		N/A		Reference	
	Weekly	658		28.97		N/A		N/A		N/A		1.07 (0.90-1.27)	
	Less than weekly	1727		78.38		N/A		N/A		N/A		1.79 (1.51-2.12)	

^a^HR: hazard ratio.

^b^N/A: not applicable.

^c^High-risk area denotes Hui county in Henan province and Pengzhou in Sichuan province in our study.

^d^High-level physical activity was defined as age-specific and sex-specific upper quarter of total physical activity level measured by metabolic equivalent of task-hours per day.

## Discussion

In a large prospective cohort study, we developed 3 nested 10-year EC absolute risk prediction models for Chinese adults aged 30-79 years. The models included age, sex, regional EC-risk level, education, family history of cancer (simple model), smoking, alcohol use, BMI (intermediate model), physical activity, hot tea consumption, and fresh fruit consumption (full model). The simple and intermediate models were externally validated in an independent prospective cohort and they exhibited excellent discrimination and good calibration. The performance of these models was compromised by keeping only predictors available in the health records but only to a small and acceptable extent.

The models that we constructed included established risk factors for EC (eg, age, smoking, alcohol use) and factors associated with increased EC risk in the CKB and in previous studies [17,18]. A previous review attributed the geographical variation in the incidence of EC in China to some unique factors in high-risk areas, such as exposure to carcinogens (eg, nitrosamines, their precursors) via water, food, and other sources [30]. To capture this variation as well as to denote some unmeasured unique factors in high-risk areas, we included regional risk level in our models. Although this predictor contributed the most to the model performance with a hazard ratio of around 6 (Table 2), our stratified validation showed that the other predictors still maintained excellent performance in both high-risk and low-risk areas (Multimedia Appendix 11).

Previous models included clinical symptoms such as dysphagia and poststernal pain to identify high-risk individuals with prevalent EC for further endoscopies [4-6]. In contrast, our models, which were intended to identify individuals at risk for developing EC in the next 10 years, did not include clinical symptoms. In a prior model developed based on a hospital-based case-control study, 25 single-nucleotide polymorphisms, in addition to age, smoking, and alcohol use, resulted in an increased AUC from 0.639 to 0.707 [7]. Some other factors such as exposure to cooking fumes, pesticides, or salty foods were also included in previous models. To develop a parsimonious model that can be potentially used widely, we did not consider genetic variants and less well-established risk factors. Nevertheless, the AUCs of our models were still higher than those of most of the previous Chinese models (range 0.681-0.843) [4-7,14].

As expected, our finding that the simple and intermediate models retained similar performance as the full model despite the fewer predictors included is reasonable since the lost information due to the removal of the predictors was more or less supplemented by other correlated predictors. A previous study showed that the discriminatory information needed for the same unit of increase of AUC exponentially increased with AUC [31]. Thus, an already high AUC of >0.8 for the simple model can only be improved by highly informative predictors. Given the similar performance and excellent discriminating ability, it is acceptable to use the simple or intermediate model in situations where the EHRs are complete and up-to-date and easily implemented in a lower-cost way than an organized screening program. Further, we noticed that the same predictors could contribute differently in subpopulations. For example, the inclusion of lifestyle factors barely improved the discriminating ability in women (Multimedia Appendix 11), which may be explained by the low prevalence and dosage intensity of smoking and alcohol use in Chinese women.

For most prediction models, underestimations or overestimations are commonly observed in an external validation, which were also observed in our study. However, across the groups defined by the deciles of predicted risks, the observed risks proportionally increased with increased predicted risk rather than an irregular misestimation. More importantly, the underestimation disappeared after recalibration. Such results implied that the predictors in our models are predictive, the coefficients estimated in the CKB are robust and generalizable, and the underestimation was mainly caused by the mismatch of EC incidence between the CKB and Changzhou cohort.

Unlike the models in previous studies, our models calculated the absolute risk of EC instead of the relative risk and could facilitate primary prevention of EC. The essence of intuition of the absolute risk can not only raise population awareness and motivate adherence to lifestyle changes but also enhance effective communication between health professionals and individuals and help health professionals identify high-risk populations for intensive lifestyle interventions. Further, several predictors in our models are modifiable, such as smoking, alcohol use, and BMI, which could be treated as targets of intervention.

Our study has several strengths. We used a large prospective cohort with the largest number of EC cases from urban, rural, high EC-risk, and low EC-risk areas in China for model development and used another prospective cohort for external validation. This method ensures that our models are robust and potentially generalizable to a wide range of areas. To the best of our knowledge, ours is the first study to develop and externally validate EC models by using 2 independent prospective Chinese cohorts. Last but not the least, we developed and validated an abbreviated version of the risk prediction model that could be easily embedded within the EHR system and enable an efficient and automatic population risk stratification. To facilitate the usage of our models, we provide an easy-to-use Stata code and example in Multimedia Appendix 16 (Stata calculator, which is a modified version of the code shared by Dr Muller) [22].

Some limitations of our study merit consideration. First, some EC cases in the CKB had only clinical diagnoses but no pathological diagnoses for various reasons. Therefore, we could not exclude cases of esophageal adenocarcinoma. However, more than 90% of the EC cases are ESCC in China [32], which was confirmed by our ongoing adjudication of incident EC cases in the CKB. More importantly, models maintained high discriminating abilities when we restricted EC cases to those with a pathological diagnosis of ESCC or those without a pathological diagnosis of ESCC. Second, we did not collect information on the family history of EC specifically and their preference for hot foods and drinks in the baseline survey. Therefore, we used the family history of any cancer and preference for very hot tea consumption as surrogates. Third, although we found limited improvement in AUC by including more predictors in the model (full model), whether other established risk factors of EC, such as disease history of the esophagus and genetic predisposition of EC could further improve the AUC warrants future research. Fourth, we only externally validated our models in low EC-risk rural areas. Further validations in other areas are warranted.

In summary, using data from 2 prospective cohorts, we developed and validated 3 nested 10-year EC absolute risk prediction models for Chinese adults, which may be particularly useful for populations in low EC-risk areas. Even the simple model with only 5 predictors available from residents’ EHRs showed excellent discrimination and good calibration, indicating its potential for broader use in tailored EC prevention. Further research is needed to assess the real-world performance in aiding population-wide stratification, identify optimal risk cutoffs for initiating intensive lifestyle interventions and endoscopy screening, and establish an optimal screening protocol (including multistage screening) for individuals or regions with different risks.

## Appendix

Multimedia Appendix 1 Supplementary methods.

Multimedia Appendix 2 Prevalence and incidence of esophageal cancer by study region.

Multimedia Appendix 3 Design of the sensitivity analysis in consideration of pathology reports.

Multimedia Appendix 4 Hazard ratios (95% CIs) for predictor variables of all-cause mortality in the China Kadoorie Biobank.

Multimedia Appendix 5 Comparison of the area under the receiver operating characteristic curve, continuous Net Reclassification Improvement, and Integrated Discrimination Improvement of esophageal cancer prediction models in the China Kadoorie Biobank and Changzhou cohort.

Multimedia Appendix 6 Comparison of the area under the receiver operating characteristic curve, continuous Net Reclassification Improvement, and Integrated Discrimination Improvement of esophageal cancer prediction models in the China Kadoorie Biobank by using bootstrap.

Multimedia Appendix 7 Age-specific observed risk of esophageal cancer in low-risk areas of the China Kadoorie Biobank and Changzhou cohort.

Multimedia Appendix 8 Discriminating ability of the recalibrated prediction models in the Changzhou cohort.

Multimedia Appendix 9 Discriminating ability of the prediction models in the China Kadoorie Biobank using data splitting.

Multimedia Appendix 10 Performance of the esophageal cancer prediction model across different predicted risk cutoffs in the China Kadoorie Biobank and Changzhou cohort.

Multimedia Appendix 11 Discrimination and calibration of the intermediate model in subcohorts of the China Kadoorie Biobank by data splitting.

Multimedia Appendix 12 Hazard ratios (95% CIs) for predictor variables of esophageal cancer prediction models developed separately in high-risk and low-risk areas of the derivation subcohort of the China Kadoorie Biobank.

Multimedia Appendix 13 Model performance of 2 esophageal cancer prediction models developed separately in high-risk and low-risk areas of the derivation subcohort of the China Kadoorie Biobank and applied in the corresponding validation subcohort.

Multimedia Appendix 14 Discriminating ability of the esophageal cancer prediction models in the China Kadoorie Biobank by data splitting in consideration of pathology reports.

Multimedia Appendix 15 Model performance of the esophageal cancer prediction models in the China Kadoorie Biobank using data splitting after excluding esophageal cancer cases occurring in the first year of follow-up.

Multimedia Appendix 16 Stata calculator.

## Abbreviations

AUC: area under the receiver operating characteristic curve
CKB: China Kadoorie Biobank
EC: esophageal cancer
EHR: electronic health record
ESCC: esophageal squamous cell carcinoma

## Data Availability

Details of how to access the China Kadoorie Biobank data and details of the data release schedule are available from [33].
