# Supplementary material for: Electronic Health Record–Based Absolute Risk Prediction Model for Esophageal Cancer in the Chinese Population: Model Development and External Validation
Source: JMIR Public Health Surveill. 2023 Mar 15;9:e43725. doi: 10.2196/43725 (PMC10132027; doi:10.2196/43725)
Supplement: Multimedia Appendix 1 [file publichealth_v9i1e43725_app1.docx]

Multimedia Appendix 1: Supplementary methods

Definition of high EC-risk areas

In China, the incidence of esophageal cancer (EC) has a large geographical variation, which was also observed in our ten study regions of the China Kadoorie Biobank (CKB) and Changzhou, the area for external validation (Multimedia Appendix 2). According to the most recent guideline for EC [19], high-risk areas for EC were defined as areas where the age-standardized incidence of EC (standardized to age structure in 2000) is greater than 15 incident cases per 100,000. Based on the age-specific incidences of EC in 2015 from the National Central Cancer Registry of China and the age structure in 2000 [8], we calculated the age-standardized incidence of EC for all Chinese and the Chinese aged 45-74, which is the recommended age range for screening [19]. The above two rates were 5.92 and 17.18 per 100,000, respectively. According to the ratio of the age-standardized rate for the Chinese aged 45-74 to all Chinese, we defined high-risk areas as areas where the age-standardized incidence of EC for the Chinese aged 45-74 is greater than 43.56 per 100,000 (15*17.18/5.92). Therefore, Hui county in Henan province and Pengzhou in Sichuan province were assigned to high-risk areas, while Changzhou was assigned to the low-risk area.

Assessment of predictors

In the CKB, sociodemographic characteristics, lifestyles, medical and family histories were collected by trained staff using a standardized laptop-based questionnaire. Anthropometric parameters (e.g., weight and height) were measured using well-calibrated instruments. Ever smokers (former and current) reported the frequency, type, and amount of tobacco smoked per day. Questions about alcohol consumption included typical drinking frequency, type of alcoholic beverage (beer, rice wine, grape wine, weak Chinese spirit with <40% alcohol content, strong Chinese spirit with ≥40% alcohol content) consumed habitually, and volume of alcohol consumed on a typical drinking day in the past 12 months. Grams of pure alcohol per day were calculated based on the above information, assuming the following alcohol content typically seen in China, beer 4%, rice wine 15%, grape wine 12%, weak spirits 38%, and strong spirits 53% [23]. For physical activity, the usual type and duration of occupational, commuting, domestic, and leisure time-related activities in the past 12 months were collected. To calculate daily total physical activity level, we multiplied the metabolic equivalent of task for each activity by the hour spent on that activity and summed the metabolic equivalent of task-hours for all activities [24]. We assessed habitual intakes of fresh vegetables, fresh fruits, red meat, and preserved vegetables in the past 12 months via a validated qualitative food frequency questionnaire [25]. We also assessed the usual frequency of tea drinking and the preference for tea temperature. BMI was calculated as weight in kilograms divided by height in meters squared.

Most predictors were assessed similarly in two cohorts, except for alcohol consumption, physical activity, and hot tea consumption. For alcohol drinking, the baseline survey of the Changzhou cohort did not distinguish between weak and strong Chinese spirits. Therefore, the grams of pure alcohol of Chinese spirits in the Changzhou cohort were calculated based on the average alcohol content of weak spirits and strong spirits (46%). Participants in Changzhou reported a preference for hot food (favorable, neutral, unfavorable) but not the hot tea. For physical activity, only the intensity of occupational physical activity and the frequency of leisure-time physical activity were assessed, which precluded the calculation of metabolic equivalent of task-hours.

Model validation

We externally validated the age-only, simple, and intermediate models, except for the full model because physical activity in MET-hours and hot tea consumption were not available in the Changzhou cohort. Internal validation was conducted using data-splitting to deal with the potential bias to be overly optimistic [26], where the model was fitted to a random two-thirds of the CKB data (derivation subcohort, n=340,097) and evaluated on the remaining one-third (validation subcohort, n=170,048). Cancer-free participants who died or lost to follow-up before 10 years or who entered the cohort after (1) Dec 31, 2007 for validation subcohort of CKB (n=32,671); (2) Jan 31, 2009 for Changzhou cohort (n=1,616) were included to test calibration but were excluded from other validation measures since it was unknown whether they could have suffered an EC if they had been followed up to 10 years.

Discrimination, the ability of a prediction model to differentiate between those who will or will not develop the outcome, was quantified by area under the receiver-operating characteristic curve (AUC), also known as c-statistics. AUC of 1.0 indicates perfect discrimination, and 0.5 indicates no discriminating value. Calibration was assessed by plotting observed risk obtained using Kaplan-Meier analyses against predicted risk by tenth of the predicted risk. Because of the large geographical variation in the incidence of EC in China, we recalibrated the models using the method proposed by the WHO CVD Risk Chart Working Group with a slight modification [21]. In brief, the recalibration process required age-specific (5-year age groups) 10-year observed risks in the low-risk areas of the CKB (*O_1_*) and Changzhou cohort (*O_2_*), which were estimated by Kaplan-Meier methods. We performed linear regressions (the formula (1)) of the transformed *O_1_* and the transformed *O_2_* to derive corresponding recalibration parameters, *b* & *k*. Age-specific observed risks of EC used to calculate *b* & *k* are shown in Multimedia Appendix 7.

$ln(-ln\boldsymbol{(}1-O_{2})) = b+k\times ln(-ln\boldsymbol{(}1-O_{1}))$…………………(1)

The recalibrated risks (*R*) were calculated using the formula (2):

$R=1-exp(-\exp\left( b+k\times ln\left( -ln\left( 1-P \right) \right) \right))$……………………(2)

Where *P* is the predicted 10-year risk of EC by the CKB models.

Reclassification statistics were used to evaluate the added predictive ability of additional predictors, including continuous Net Reclassification Improvement and Integrated Discrimination Improvement. In the internal validation, we also used a 500-sample bootstrapping to overcome overfitting when estimating the AUC, continuous Net Reclassification Improvement, and Integrated Discrimination Improvement. This internal validation is a less biased method than data-splitting, but extremely time-consuming in our large dataset and can not draw receiver-operating characteristics curve and calibration plot. In each bootstrapping sample, the absolute risk model was reconstructed and then validated. In the internal validation, calibration and discrimination were also assessed in subgroups defined by regional EC-risk level (high/low-risk), residence area (urban/rural), sex (male/female), age group (30-49/50-64/65-79 years), and special populations of aged 65 years and older or with diabetes or hypertension (yes/no) who are of particular concern to the Basic Public Health Service.

To offer a reference for appropriate cut-offs for primary care practices, we estimated a range of performance indices corresponding to a series of cut-offs. The indices included: percent of high-risk population, sensitivity, specificity, Youden’s index (sensitivity + specificity - 1), positive/negative predictive value, numbers needed to screen (one divided by the positive predictive value) to confirm one case in the next 10 years, and missed cases per confirmed case (sensitivity divided by false-negative rate).

All statistical analyses were performed using Stata (version 15, StataCorp). We modified the Stata codes that were shared by Dr. Muller to calculate the 10-year absolute risk of EC in the presence of EC [22]. Reporting the study adhered to the TRIPOD (Transparent Reporting of a Multivariable Prediction Model for Individual Prognosis or Diagnosis) statement [26].
