# Supplementary material for: Electronic Health Record–Based Absolute Risk Prediction Model for Esophageal Cancer in the Chinese Population: Model Development and External Validation
Source: JMIR Public Health Surveill. 2023 Mar 15;9:e43725. doi: 10.2196/43725 (PMC10132027; doi:10.2196/43725)
Supplement: Multimedia Appendix 2 [file publichealth_v9i1e43725_app2.docx]

Multimedia Appendix 2: Prevalence and incidence of esophageal cancer by study region

|  | China Kadoorie Biobank | | | | | | | | | | | Changzhou | Nation in 2015 |
| --- | --- | --- | --- | --- | --- | --- | --- | --- | --- | --- | --- | --- | --- |
|  | Shandong | Heilongjiang | Hainan | Jiangsu | Guangxi | Sichuan | Gansu | Henan | Zhejiang | Hunan | Total |  |  |
|  | Qingdao | Harbin | Haikou | Suzhou | Liuzhou | Pengzhou | Maiji | Hui county | Tongxiang | Liuyang |  |  |  |
| No. of participants, n | 35,507 | 57,556 | 29,686 | 53,269 | 50,174 | 55,686 | 49,887 | 63,356 | 57,704 | 59,898 | 512,723 | 18,670 | / |
| Prevalent EC cases at baseline | |  |  |  |  |  |  |  |  |  |  |  |  |
| No., n | 3 | 3 | 3 | 15 | 7 | 18 | 2 | 233 | 9 | 3 | 296 | 17 | / |
| Prevalence, ‰ | 0.08 | 0.05 | 0.1 | 0.28 | 0.14 | 0.32 | 0.04 | 3.68 | 0.16 | 0.05 | 0.58 | 0.09 | / |
| Incidence of EC cases^a^ |  |  |  |  |  |  |  |  |  |  |  |  |  |
| Age groups^b^ |  |  |  |  |  |  |  |  |  |  |  |  |  |
| 0-4 | / | / | / | / | / | / | / | / | / | / | / | / | 0.02 |
| 5- | / | / | / | / | / | / | / | / | / | / | / | / | 0.00 |
| 10- | / | / | / | / | / | / | / | / | / | / | / | / | 0.01 |
| 15- | / | / | / | / | / | / | / | / | / | / | / | / | 0.01 |
| 20- | / | / | / | / | / | / | / | / | / | / | / | / | 0.04 |
| 25- | / | / | / | / | / | / | / | / | / | / | / | / | 0.10 |
| 30- | 0.00 | 0.00 | 0.00 | 0.00 | 0.00 | 0.00 | 0.00 | 0.00 | 0.00 | 0.00 | 0.00 | 27.10 | 0.17 |
| 35- | 0.00 | 0.00 | 0.00 | 0.00 | 0.00 | 3.74 | 0.00 | 6.82 | 0.00 | 4.66 | 1.94 | 0.00 | 0.38 |
| 40- | 2.32 | 0.00 | 0.00 | 1.65 | 0.00 | 6.98 | 3.75 | 7.19 | 1.85 | 1.42 | 3.04 | 3.84 | 0.85 |
| 45- | 7.54 | 5.97 | 4.65 | 8.44 | 6.04 | 17.89 | 2.21 | 28.10 | 2.15 | 3.94 | 9.51 | 0.00 | 2.42 |
| 50- | 15.11 | 10.29 | 4.68 | 12.51 | 1.28 | 34.38 | 10.97 | 68.33 | 4.68 | 9.86 | 19.10 | 14.47 | 4.74 |
| 55- | 29.58 | 11.96 | 2.36 | 36.24 | 2.22 | 78.32 | 26.21 | 168.80 | 9.98 | 17.13 | 42.65 | 49.21 | 11.48 |
| 60- | 18.52 | 14.71 | 12.80 | 41.27 | 13.09 | 84.49 | 47.93 | 250.59 | 22.18 | 18.77 | 58.27 | 54.85 | 26.04 |
| 65- | 33.63 | 17.79 | 18.05 | 56.78 | 20.28 | 121.56 | 66.17 | 423.29 | 42.23 | 33.32 | 88.64 | 101.17 | 43.87 |
| 70- | 67.94 | 11.37 | 9.82 | 91.96 | 21.99 | 165.18 | 99.80 | 581.15 | 42.28 | 37.87 | 108.45 | 119.90 | 56.89 |
| 75- | 117.84 | 22.99 | 52.47 | 115.20 | 32.65 | 162.39 | 129.73 | 682.15 | 62.62 | 60.57 | 129.19 | 58.69 | 69.91 |
| 80- | 77.74 | 14.07 | 11.71 | 203.04 | 25.65 | 197.61 | 45.70 | 894.30 | 84.06 | 77.81 | 150.35 | 191.83 | 84.33 |
| 85- | 0.00 | 0.00 | 0.00 | 173.27 | 0.00 | 487.85 | 229.42 | 783.86 | 129.67 | 0.00 | 154.51 | 166.09 | 76.74 |
| Crude | 24.68 | 10.99 | 10.27 | 37.57 | 10.58 | 67.83 | 28.09 | 189.07 | 18.97 | 18.14 | 46.17 | 47.12 | 17.87 |
| Age-standardized for 45-74^c^ | 22.38 | 10.90 | 7.46 | 31.09 | 8.46 | 64.97 | 30.11 | 183.65 | 14.86 | 15.70 | 41.33 | 40.61 | 17.18 |

EC, esophageal cancer

^a^Incidence of EC was calculated in participants without a previous diagnosis of cancer and was given as number of cases per 100,000 person-years.

^b^Age groups were defined according to attained age.

^c^Age-standardized incidence of EC for the Chinese aged 45-74 was standardized to the age structure in China in 2000.
