# Supplementary material for: Electronic Health Record–Based Absolute Risk Prediction Model for Esophageal Cancer in the Chinese Population: Model Development and External Validation
Source: JMIR Public Health Surveill. 2023 Mar 15;9:e43725. doi: 10.2196/43725 (PMC10132027; doi:10.2196/43725)
Supplement: Multimedia Appendix 3 [file publichealth_v9i1e43725_app3.docx]

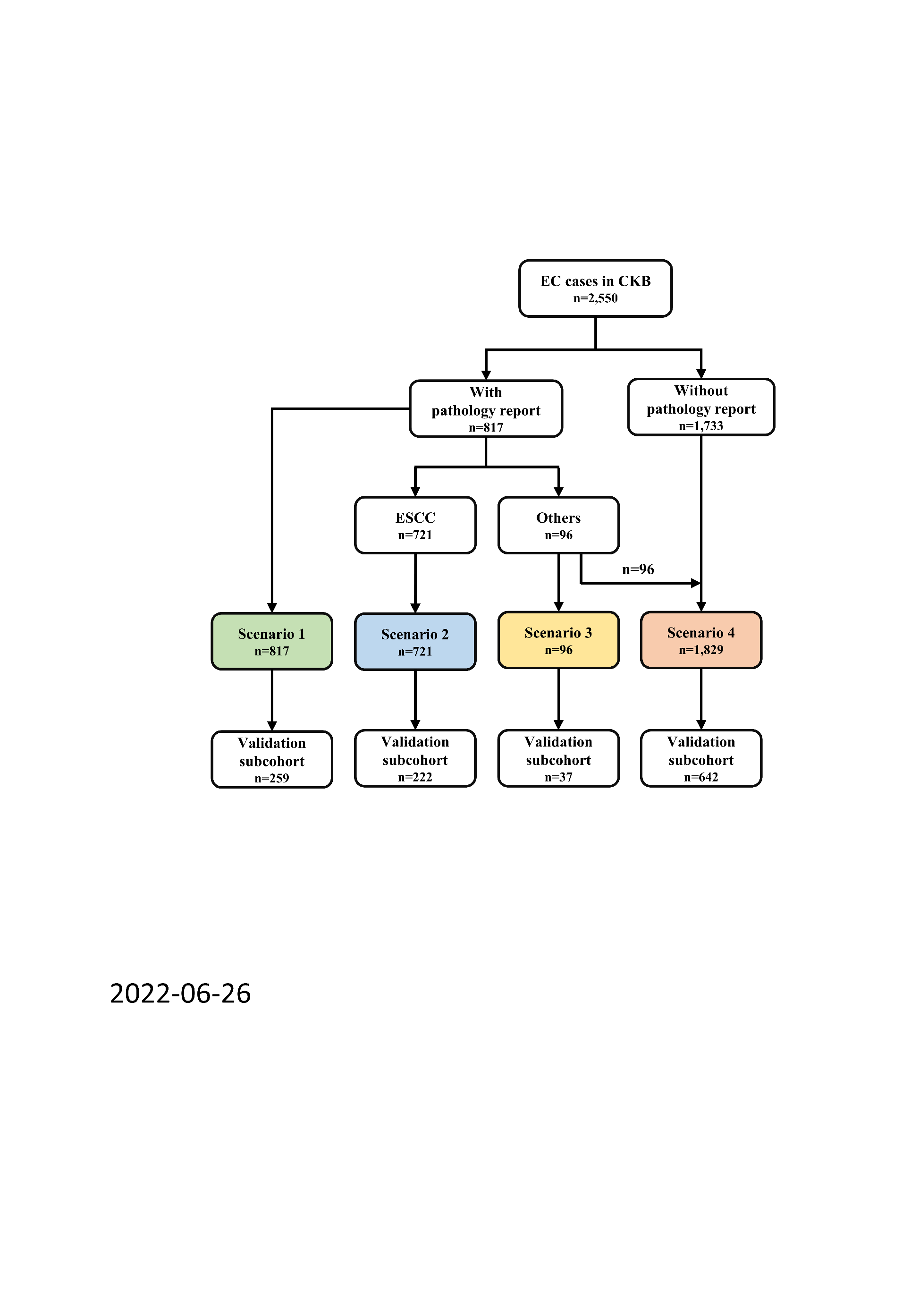


Multimedia Appendix 3: Design of the sensitivity analysis in consideration of pathology reports.

CKB, China Kadoorie Biobank; EC, esophageal cancer; ESCC, esophageal squamous cell carcinoma.

Among 2,550 incident EC cases during follow-up of 510,145 cancer-free participants, 817 were pathologically confirmed. Among them, 721 cases were pathologically confirmed as ESCC and 96 cases were not.

In scenario 1, all cases were pathologically confirmed. In scenario 2, all cases were pathologically confirmed as ESCC. In scenario 3, all cases were pathologically confirmed, but not as ESCC. In scenario 4, all cases were not pathologically confirmed or were pathologically confirmed, but not as ESCC. In the analysis of each scenario, EC cases that did not meet the corresponding criteria were excluded.

And models were fitted to a random two-thirds of the CKB data (derivation subcohort) and evaluated on the remaining one-third (validation subcohort).
