# Supplementary material for: Electronic Health Record–Based Absolute Risk Prediction Model for Esophageal Cancer in the Chinese Population: Model Development and External Validation
Source: JMIR Public Health Surveill. 2023 Mar 15;9:e43725. doi: 10.2196/43725 (PMC10132027; doi:10.2196/43725)
Supplement: Multimedia Appendix 4 [file publichealth_v9i1e43725_app4.docx]

Multimedia Appendix 4: HRs (95% CIs) for predictor variables of all-cause mortality in China Kadoorie Biobank

|  | Cases | Cases/PYs | HR (95% CI) |
| --- | --- | --- | --- |
|  |  | (1/100,000) |  |
| Constant |  |  | 0.14 (0.13-0.14) |
| Spline basis of age (knots:30, 60, 70, 90) | |  |  |
| First |  |  | 2.62 (2.49-2.76) |
| Second |  |  | 0.88 (0.87-0.90) |
| Third |  |  | 0.96 (0.96-0.97) |
| Sex |  |  |  |
| Male | 27,641 | 1,242.95 | Reference |
| Female | 20,948 | 634.00 | 0.58 (0.57-0.59) |
| Residence area | |  |  |
| Rural | 30,307 | 974.53 | Reference |
| Urban | 18,282 | 756.07 | 0.67 (0.66-0.68) |
| High-risk area^a^ | |  |  |
| No | 35,906 | 845.95 | Reference |
| Yes | 12,683 | 988.18 | 1.07 (1.05-1.10) |

HR, hazard ratio; CI, confidence interval; PYs, person-years.

^a^High-risk area denotes Hui county, Henan province, and Pengzhou, Sichuan province in our study.
