# Supplementary material for: Electronic Health Record–Based Absolute Risk Prediction Model for Esophageal Cancer in the Chinese Population: Model Development and External Validation
Source: JMIR Public Health Surveill. 2023 Mar 15;9:e43725. doi: 10.2196/43725 (PMC10132027; doi:10.2196/43725)
Supplement: Multimedia Appendix 5 [file publichealth_v9i1e43725_app5.docx]

Multimedia Appendix 5: Comparison of the area under the receiver operating characteristic curve, continuous Net Reclassification Improvement, and Integrated Discrimination Improvement of esophageal cancer prediction models in the China Kadoorie Biobank and Changzhou cohort^b^.

| Comparison | No. | Difference in AUC | |  | cNRI (95% CI) | |  | IDI | |
| --- | --- | --- | --- | --- | --- | --- | --- | --- | --- |
|  |  | Point estimate | *P* value |  | Case (%) | Non-case (%) |  | Point estimate | *P* value |
| **China Kadoorie Biobank** |  |  |  |  |  |  |  |  |  |
| Simple – Age-only^c^ | 137,377 | 0.1233 | <0.001 |  | 39.0 (31.3 to 46.7) | 51.7 (51.2 to 52.2) |  | 0.0126 | <0.001 |
| Intermediate – Simple^c^ | 137,377 | 0.0084 | <0.001 |  | -2.8 (-10.5 to 4.9) | 12.4 (11.9 to 12.9) |  | 0.0015 | <0.001 |
| Full – Intermediate^c^ | 137,377 | 0.0035 | 0.014 |  | 29.7 (22.0 to 37.4) | 17.4 (16.8 to 17.9) |  | 0.0013 | <0.001 |
| High-risk – Full^d^ | 31,672 | <0.0001 | 0.999 |  | 21.0 (11.6 to 30.4) | -30.0 (-31.1 to -28.9) |  | -0.0008 | 0.011 |
| Low-risk – Full^e^ | 105,705 | 0.0144 | <0.001 |  | 13.6 (0.2 to 27.0) | 25.8 (25.2 to 26.4) |  | 0.0012 | <0.001 |
| **Changzhou cohort** |  |  |  |  |  |  |  |  |  |
| Simple – Age-only^f^ | 16,836 | 0.0499 | 0.003 |  | -79.5 (-101.7 to -57.3) | 93.1 (91.5 to 94.6) |  | -0.00063 | 0.056 |
| Intermediate – Simple^f^ | 16,836 | 0.0074 | 0.243 |  | 33.3 (11.1 to 55.5) | -9.0 (-10.5 to -7.5) |  | 0.0010 | 0.001 |

AUC, area under the receiver operating characteristic curve; cNRI, continuous net reclassification index; IDI, integrated discrimination index.

^a^The models included age (age-only model), sex, regional risk level, education, family history of cancer (above predictors: simple model), smoking, alcohol drinking, BMI (intermediate model), physical activity, hot tea consumption, and fresh fruit consumption (full model).

^b^Internal validation was conducted in the China Kadoorie Biobank using data-splitting, and external validation was conducted in the Changzhou cohort.

^c^Comparison was conducted in the whole validation subcohort.

^d^High-risk model was developed and internally validated in high-risk areas (Hui county, Henan province, and Pengzhou, Sichuan province).

^e^Low-risk model was developed and internally validated in low-risk areas (other study regions).

^f^Comparison was conducted among participants in the Changzhou cohort.
