# Supplementary material for: Electronic Health Record–Based Absolute Risk Prediction Model for Esophageal Cancer in the Chinese Population: Model Development and External Validation
Source: JMIR Public Health Surveill. 2023 Mar 15;9:e43725. doi: 10.2196/43725 (PMC10132027; doi:10.2196/43725)
Supplement: Multimedia Appendix 6 [file publichealth_v9i1e43725_app6.docx]

Multimedia Appendix 6: Comparison of the area under the receiver operating characteristic curve, continuous Net Reclassification Improvement, and Integrated Discrimination Improvement of esophageal cancer prediction models^a^ in the China Kadoorie Biobank using bootstrap.

| Comparison | No. | AUC | Difference in AUC | |  | cNRI (95% CI) | |  | IDI | |
| --- | --- | --- | --- | --- | --- | --- | --- | --- | --- | --- |
|  |  |  | Point estimate | *P* value |  | Case (%) | Non-case (%) |  | Point estimate | *P* value |
| Age-only | 510,415 | 0.754 (0.745,0.763) |  |  |  |  |  |  |  |  |
| Simple^b^ | 510,415 | 0.870 (0.862,0.878) | 0.1159 | <0.001 |  | 36.54 (32.34 to 40.75) | 51.93 (49.92 to 53.94) |  | 0.0127 | <0.001 |
| Intermediate^b^ | 510,415 | 0.877 (0.870,0.884) | 0.0070 | <0.001 |  | -13.09 (-32.26 to 6.08) | 13.66 (-3.31 to 30.63) |  | 0.0013 | <0.001 |
| Full^b^ | 510,415 | 0.879 (0.872,0.887) | 0.0026 | 0.002 |  | 26.07 (11.35 to 40.80) | 18.69 (9.83 to 27.55) |  | 0.0013 | <0.001 |
| High-risk^c^ | 118,407 | 0.831 (0.820,0.841) | 0.0032 | 0.029 |  | 22.92 (10.49 to 35.34) | -32.87 (-60.86 to -4.88) |  | -0.0008 | 0.067 |
| Low-risk^d^ | 391,738 | 0.847 (0.834,0.860) | 0.0068 | 0.018 |  | 3.63 (-8.32 to 15.57) | 31.90 (6.41 to 57.39) |  | 0.0012 | <0.001 |

AUC, area under the receiver operating characteristic curve; cNRI, continuous net reclassification index; IDI, integrated discrimination index.

^a^The models included age (age-only model), sex, regional risk level, education, family history of cancer (above predictors: simple model), smoking, alcohol drinking, BMI (intermediate model), physical activity, hot tea consumption, and fresh fruit consumption (full model).

^b^Model was compared with a more parsimonious model (e.g., compared with the simple model, the AUC of the intermediate model increased by 0.007), unless otherwise stated

^c^High-risk model was developed and internally validated in high-risk areas (Hui county, Henan province and Pengzhou, Sichuan province).

^d^Low-risk model was developed and internally validated in low-risk areas (other study regions).
