# Supplementary material for: Electronic Health Record–Based Absolute Risk Prediction Model for Esophageal Cancer in the Chinese Population: Model Development and External Validation
Source: JMIR Public Health Surveill. 2023 Mar 15;9:e43725. doi: 10.2196/43725 (PMC10132027; doi:10.2196/43725)
Supplement: Multimedia Appendix 7 [file publichealth_v9i1e43725_app7.docx]

Multimedia Appendix 7: Age-specific observed risk of esophageal cancer in low-risk areas of China Kadoorie Biobank and Changzhou cohort

|  | Low-risk areas of China Kadoorie Biobank | |  | Changzhou cohort | |
| --- | --- | --- | --- | --- | --- |
|  | No. | *O_1_* |  | No. | *O_2_* |
| 30- | 7,276 | 0.00 |  | 1,670 | 6.01 |
| 35- | 49,419 | 1.64 |  | 2,211 | 0.00 |
| 40- | 63,948 | 4.46 |  | 2,501 | 4.01 |
| 45- | 53,552 | 10.51 |  | 2,468 | 16.67 |
| 50- | 66,275 | 16.55 |  | 2,809 | 36.71 |
| 55- | 53,219 | 23.76 |  | 2,497 | 99.70 |
| 60- | 38,042 | 35.31 |  | 1,595 | 88.29 |
| 65- | 33,481 | 47.06 |  | 1,214 | 88.77 |
| 70- | 23,705 | 69.24 |  | 797 | 123.36 |
| 75- | 2,821 | 65.61 |  | 679 | 125.10 |

*O_1_* and *O_1_* refer to age-specific (5-year age groups) 10-year observed risks per 10,000 in the low-risk areas of the CKB and Changzhou cohort, respectively, which were estimated by Kaplan-Meier methods
