# Supplementary material for: Electronic Health Record–Based Absolute Risk Prediction Model for Esophageal Cancer in the Chinese Population: Model Development and External Validation
Source: JMIR Public Health Surveill. 2023 Mar 15;9:e43725. doi: 10.2196/43725 (PMC10132027; doi:10.2196/43725)
Supplement: Multimedia Appendix 8 [file publichealth_v9i1e43725_app8.docx]

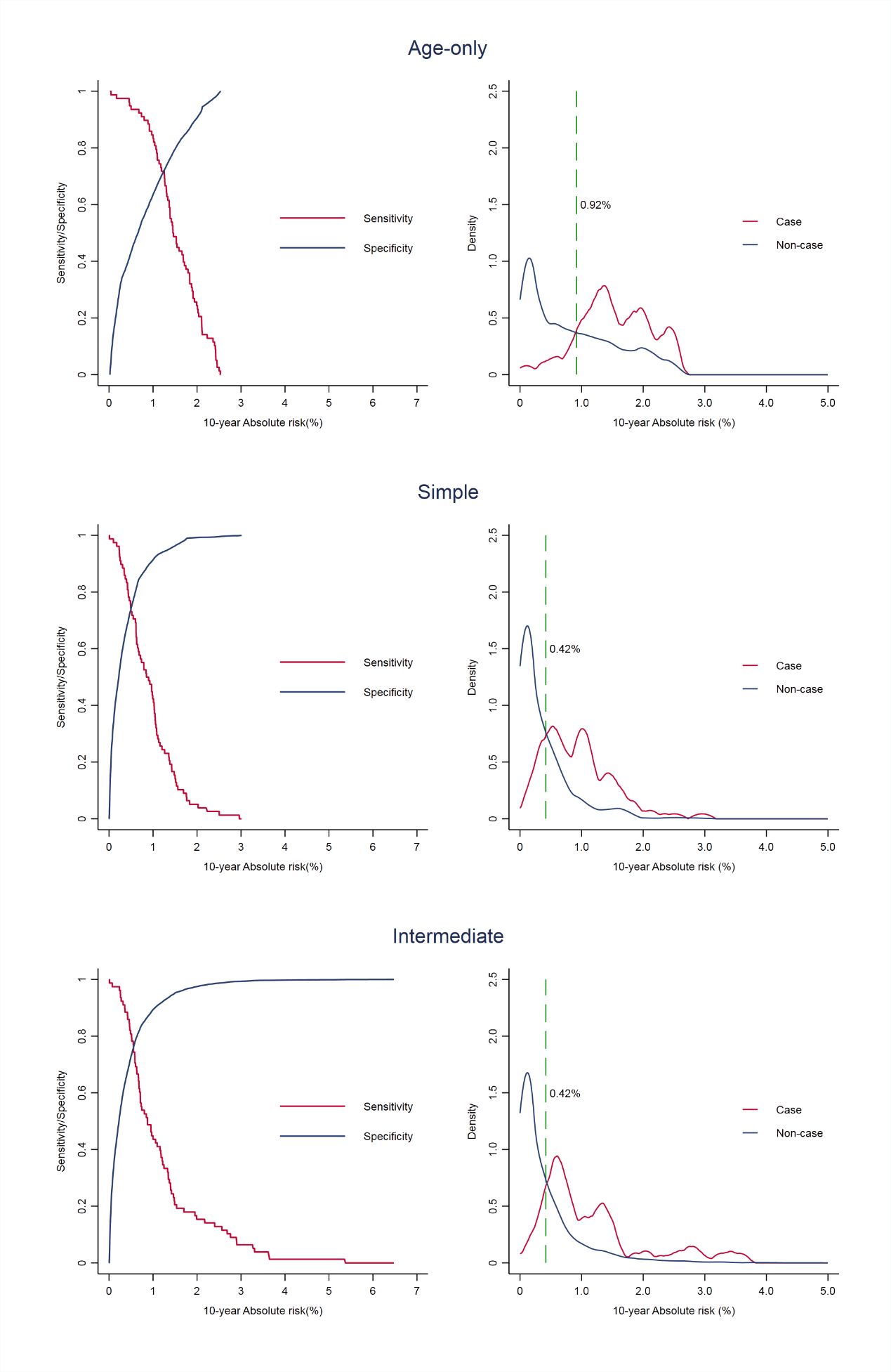


Multimedia Appendix 8: Discriminating ability of the recalibrated prediction models in Changzhou cohort.

Sensitivity and specificity based on the recalibrated 10-year predicted risk cut-offs (left); the distribution of the recalibrated predicted risk for both cases and non-cases of esophageal cancer (right).

Models were fitted in the whole China Kadoorie Biobank data and evaluated on the Changzhou cohort.

Models were recalibrated using the method proposed by the WHO CVD Risk Chart Working Group. For details see Multimedia Appendix 1.

.
