# Supplementary material for: Electronic Health Record–Based Absolute Risk Prediction Model for Esophageal Cancer in the Chinese Population: Model Development and External Validation
Source: JMIR Public Health Surveill. 2023 Mar 15;9:e43725. doi: 10.2196/43725 (PMC10132027; doi:10.2196/43725)
Supplement: Multimedia Appendix 9 [file publichealth_v9i1e43725_app9.docx]

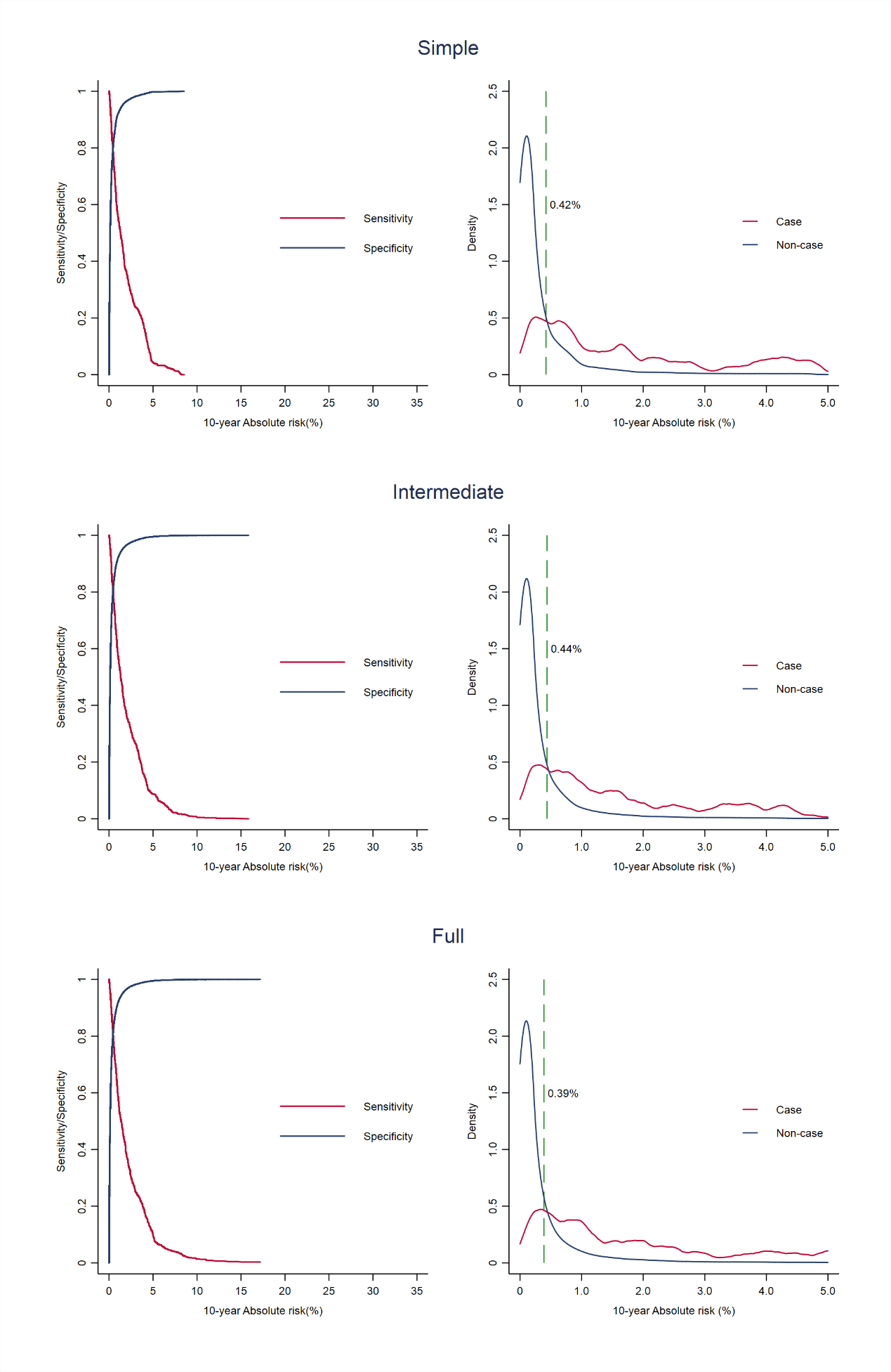


Multimedia Appendix 9: Discriminating ability of the prediction models in China Kadoorie Biobank using data-splitting.

Sensitivity and specificity based on the 10-year predicted risk cut-offs (left); the distribution of the predicted risk for both cases and non-cases of esophageal cancer (right).

Models were fitted to a random two-thirds of the China Kadoorie Biobank data and evaluated on the remaining one-third.

The green dash line indicates the cut-off, where Youden’s index reaches the maximum.
