# Supplementary material for: Electronic Health Record–Based Absolute Risk Prediction Model for Esophageal Cancer in the Chinese Population: Model Development and External Validation
Source: JMIR Public Health Surveill. 2023 Mar 15;9:e43725. doi: 10.2196/43725 (PMC10132027; doi:10.2196/43725)
Supplement: Multimedia Appendix 10 [file publichealth_v9i1e43725_app10.docx]

Multimedia Appendix 10-1: Performance of the recalibrated esophageal cancer prediction model across different predicted risk cut-offs of the simple model in Changzhou cohort.

| Cut-off (%) | Percent of high-risk population (%)a | Sensitivity (%) | Specificity (%) | Youden's index (%) | Positive predictive value (%) | Negative predictive value (%) | Number needed to be screened to confirm one case | Missed cases per confirmed case |
| --- | --- | --- | --- | --- | --- | --- | --- | --- |
| 0.1 | 58.45 | 97.44 | 41.74 | 39.18 | 0.77 | 99.97 | 129.39 | 0.03 |
| 0.2 | 44.82 | 91.03 | 55.39 | 46.42 | 0.94 | 99.92 | 106.23 | 0.10 |
| 0.3 | 34.87 | 84.62 | 65.36 | 49.98 | 1.12 | 99.89 | 88.89 | 0.18 |
| 0.4 | 27.85 | 76.92 | 72.38 | 49.30 | 1.28 | 99.85 | 78.10 | 0.30 |
| 0.5 | 22.24 | 70.51 | 77.99 | 48.50 | 1.47 | 99.82 | 68.04 | 0.42 |
| 0.6 | 16.72 | 60.26 | 83.48 | 43.74 | 1.67 | 99.78 | 59.87 | 0.66 |
| 0.7 | 14.07 | 55.13 | 86.12 | 41.25 | 1.82 | 99.76 | 55.07 | 0.81 |
| 0.8 | 12.30 | 52.56 | 87.89 | 40.45 | 1.98 | 99.75 | 50.49 | 0.90 |
| 0.9 | 10.28 | 48.72 | 89.90 | 38.62 | 2.20 | 99.74 | 45.53 | 1.05 |
| 1.0 | 8.84 | 42.31 | 91.32 | 33.63 | 2.22 | 99.71 | 45.06 | 1.36 |
| 1.1 | 7.32 | 30.77 | 92.79 | 23.56 | 1.95 | 99.65 | 51.33 | 2.25 |
| 1.2 | 6.45 | 25.64 | 93.64 | 19.28 | 1.84 | 99.63 | 54.25 | 2.90 |
| 1.3 | 5.82 | 24.36 | 94.26 | 18.62 | 1.94 | 99.63 | 51.58 | 3.11 |
| 1.4 | 5.34 | 23.08 | 94.75 | 17.83 | 2.00 | 99.62 | 49.89 | 3.33 |
| 1.5 | 4.67 | 19.23 | 95.40 | 14.63 | 1.91 | 99.61 | 52.40 | 4.20 |
| 1.6 | 3.99 | 16.67 | 96.07 | 12.74 | 1.94 | 99.60 | 51.62 | 5.00 |
| 1.7 | 3.27 | 11.54 | 96.76 | 8.30 | 1.63 | 99.58 | 61.22 | 7.67 |
| 1.8 | 2.64 | 10.26 | 97.39 | 7.65 | 1.80 | 99.57 | 55.63 | 8.75 |
| 1.9 | 1.97 | 8.97 | 98.06 | 7.03 | 2.11 | 99.57 | 47.43 | 10.14 |
| 2.0 | 1.05 | 6.41 | 98.98 | 5.39 | 2.84 | 99.56 | 35.20 | 14.60 |

Simple model was recalibrated using the method proposed by the WHO CVD Risk Chart Working Group with a slight modification. For details see Multimedia Appendix 1: Supplementary methods.

^a^High-risk population is defined as people whose 10-year predicted risk are higher than the corresponding cut-off.

Multimedia Appendix 10-2: Performance of the recalibrated esophageal cancer prediction model across different predicted risk cut-offs of the intermediate model in Changzhou cohort.

| Cut-off (%) | Percent of high-risk population (%)a | Sensitivity (%) | Specificity (%) | Youden's index (%) | Positive predictive value (%) | Negative predictive value (%) | Number needed to be screened to confirm one case | Missed cases per confirmed case |
| --- | --- | --- | --- | --- | --- | --- | --- | --- |
| 0.1 | 59.47 | 97.44 | 40.70 | 38.14 | 0.76 | 99.97 | 131.67 | 0.03 |
| 0.2 | 45.53 | 93.59 | 54.69 | 48.28 | 0.95 | 99.95 | 104.95 | 0.07 |
| 0.3 | 36.21 | 88.46 | 64.03 | 52.49 | 1.13 | 99.92 | 88.30 | 0.13 |
| 0.4 | 29.47 | 83.33 | 70.78 | 54.11 | 1.31 | 99.89 | 76.29 | 0.20 |
| 0.5 | 23.77 | 74.36 | 76.46 | 50.82 | 1.45 | 99.84 | 68.97 | 0.34 |
| 0.6 | 19.34 | 66.67 | 80.88 | 47.55 | 1.60 | 99.81 | 62.58 | 0.50 |
| 0.7 | 16.23 | 53.85 | 83.94 | 37.79 | 1.54 | 99.74 | 65.02 | 0.86 |
| 0.8 | 14.35 | 52.56 | 85.83 | 38.39 | 1.70 | 99.74 | 58.90 | 0.90 |
| 0.9 | 12.60 | 48.72 | 87.57 | 36.29 | 1.79 | 99.73 | 55.79 | 1.05 |
| 1.0 | 10.83 | 44.87 | 89.32 | 34.19 | 1.92 | 99.71 | 52.09 | 1.23 |
| 1.1 | 9.64 | 42.31 | 90.51 | 32.82 | 2.03 | 99.70 | 49.15 | 1.36 |
| 1.2 | 8.66 | 41.03 | 91.49 | 32.52 | 2.20 | 99.70 | 45.53 | 1.44 |
| 1.3 | 7.68 | 34.62 | 92.45 | 27.07 | 2.09 | 99.67 | 47.85 | 1.89 |
| 1.4 | 6.81 | 33.33 | 93.31 | 26.64 | 2.27 | 99.67 | 44.08 | 2.00 |
| 1.5 | 5.97 | 28.21 | 94.14 | 22.35 | 2.19 | 99.65 | 45.64 | 2.55 |
| 1.6 | 5.22 | 23.08 | 94.87 | 17.95 | 2.05 | 99.62 | 48.78 | 3.33 |
| 1.7 | 4.53 | 19.23 | 95.54 | 14.77 | 1.97 | 99.61 | 50.80 | 4.20 |
| 1.8 | 4.21 | 19.23 | 95.86 | 15.09 | 2.12 | 99.61 | 47.20 | 4.20 |
| 1.9 | 3.78 | 19.23 | 96.29 | 15.52 | 2.36 | 99.61 | 42.40 | 4.20 |
| 2.0 | 3.37 | 17.95 | 96.70 | 14.65 | 2.47 | 99.61 | 40.50 | 4.57 |

Intermediate model was recalibrated using the method proposed by the WHO CVD Risk Chart Working Group with a slight modification. For details see Multimedia Appendix 1: Supplementary methods.

^a^High-risk population is defined as people whose 10-year predicted risk are higher than the corresponding cut-off.

Multimedia Appendix 10-3: Performance of the esophageal cancer prediction model across different predicted risk cut-offs of the simple model in China Kadoorie Biobank using data-splitting.

| Cut-off (%) | Percent of high-risk population (%)^a^ | Sensitivity (%) | Specificity (%) | Youden's index (%) | Positive predictive value (%) | Negative predictive value (%) | Number needed to be screened to confirm one case | Missed cases per confirmed case |
| --- | --- | --- | --- | --- | --- | --- | --- | --- |
| 0.1 | 58.81 | 96.90 | 41.37 | 38.27 | 0.77 | 99.96 | 129.07 | 0.03 |
| 0.2 | 39.53 | 91.80 | 60.72 | 52.52 | 1.09 | 99.94 | 91.58 | 0.09 |
| 0.3 | 28.46 | 86.07 | 71.81 | 57.88 | 1.42 | 99.91 | 70.31 | 0.16 |
| 0.4 | 22.79 | 81.42 | 77.49 | 58.91 | 1.68 | 99.89 | 59.51 | 0.23 |
| 0.5 | 18.66 | 77.24 | 81.62 | 58.86 | 1.95 | 99.87 | 51.36 | 0.29 |
| 0.6 | 15.78 | 72.29 | 84.49 | 56.78 | 2.15 | 99.85 | 46.42 | 0.38 |
| 0.7 | 13.26 | 67.80 | 87.00 | 54.80 | 2.40 | 99.83 | 41.58 | 0.47 |
| 0.8 | 10.85 | 62.85 | 89.39 | 52.24 | 2.72 | 99.80 | 36.72 | 0.59 |
| 0.9 | 9.34 | 59.13 | 90.90 | 50.03 | 2.98 | 99.79 | 33.57 | 0.69 |
| 1.0 | 8.40 | 56.66 | 91.83 | 48.49 | 3.17 | 99.78 | 31.52 | 0.77 |
| 1.1 | 7.66 | 54.18 | 92.56 | 46.74 | 3.33 | 99.77 | 30.05 | 0.85 |
| 1.2 | 6.98 | 52.01 | 93.23 | 45.24 | 3.50 | 99.76 | 28.55 | 0.92 |
| 1.3 | 6.34 | 50.00 | 93.86 | 43.86 | 3.71 | 99.75 | 26.98 | 1.00 |
| 1.4 | 5.77 | 47.83 | 94.43 | 42.26 | 3.90 | 99.74 | 25.64 | 1.09 |
| 1.5 | 5.29 | 46.28 | 94.90 | 41.18 | 4.11 | 99.73 | 24.32 | 1.16 |
| 1.6 | 4.85 | 43.65 | 95.33 | 38.98 | 4.23 | 99.72 | 23.62 | 1.29 |
| 1.7 | 4.44 | 41.02 | 95.74 | 36.76 | 4.35 | 99.71 | 23.00 | 1.44 |
| 1.8 | 4.11 | 37.93 | 96.05 | 33.98 | 4.34 | 99.70 | 23.04 | 1.64 |
| 1.9 | 3.87 | 37.00 | 96.29 | 33.29 | 4.50 | 99.69 | 22.22 | 1.70 |
| 2.0 | 3.67 | 35.60 | 96.49 | 32.09 | 4.57 | 99.69 | 21.89 | 1.81 |

Simple model was fitted to a random two-thirds of the China Kadoorie Biobank data and evaluated on the remaining one-third.

^a^High-risk population is defined as people whose 10-year predicted risk are higher than the corresponding cut-off.

Multimedia Appendix 10-4: Performance of the esophageal cancer prediction model across different predicted risk cut-offs of the intermediate model in the China Kadoorie Biobank using data-splitting.

| Cut-off (%) | Percent of high-risk population (%)^a^ | Sensitivity (%) | Specificity (%) | Youden's index (%) | Positive predictive value (%) | Negative predictive value (%) | Number needed to be screened to confirm one case | Missed cases per confirmed case |
| --- | --- | --- | --- | --- | --- | --- | --- | --- |
| 0.1 | 58.30 | 96.75 | 41.88 | 38.63 | 0.78 | 99.96 | 128.15 | 0.03 |
| 0.2 | 39.01 | 92.72 | 61.25 | 53.97 | 1.12 | 99.94 | 89.46 | 0.08 |
| 0.3 | 28.20 | 86.53 | 72.07 | 58.60 | 1.44 | 99.91 | 69.31 | 0.16 |
| 0.4 | 22.19 | 82.66 | 78.10 | 60.76 | 1.75 | 99.90 | 57.08 | 0.21 |
| 0.5 | 18.22 | 78.33 | 82.07 | 60.40 | 2.02 | 99.88 | 49.46 | 0.28 |
| 0.6 | 15.21 | 74.46 | 85.07 | 59.53 | 2.30 | 99.86 | 43.43 | 0.34 |
| 0.7 | 12.66 | 70.12 | 87.61 | 57.73 | 2.60 | 99.84 | 38.40 | 0.43 |
| 0.8 | 10.83 | 65.79 | 89.43 | 55.22 | 2.86 | 99.82 | 35.00 | 0.52 |
| 0.9 | 9.59 | 62.54 | 90.66 | 53.20 | 3.07 | 99.81 | 32.62 | 0.60 |
| 1.0 | 8.47 | 58.20 | 91.76 | 49.96 | 3.23 | 99.79 | 30.95 | 0.72 |
| 1.1 | 7.64 | 55.73 | 92.59 | 48.32 | 3.43 | 99.77 | 29.16 | 0.79 |
| 1.2 | 6.90 | 53.25 | 93.32 | 46.57 | 3.63 | 99.76 | 27.56 | 0.88 |
| 1.3 | 6.26 | 50.93 | 93.96 | 44.89 | 3.83 | 99.75 | 26.12 | 0.96 |
| 1.4 | 5.72 | 48.61 | 94.48 | 43.09 | 3.99 | 99.74 | 25.04 | 1.06 |
| 1.5 | 5.26 | 47.06 | 94.94 | 42.00 | 4.21 | 99.74 | 23.77 | 1.13 |
| 1.6 | 4.81 | 43.19 | 95.37 | 38.56 | 4.22 | 99.72 | 23.68 | 1.32 |
| 1.7 | 4.46 | 41.18 | 95.71 | 36.89 | 4.34 | 99.71 | 23.04 | 1.43 |
| 1.8 | 4.13 | 39.47 | 96.04 | 35.51 | 4.49 | 99.70 | 22.25 | 1.53 |
| 1.9 | 3.82 | 38.39 | 96.34 | 34.73 | 4.72 | 99.70 | 21.17 | 1.60 |
| 2.0 | 3.57 | 36.22 | 96.58 | 32.80 | 4.76 | 99.69 | 20.99 | 1.76 |

Intermediate model was fitted to a random two-thirds of the China Kadoorie Biobank data and evaluated on the remaining one-third.

^a^High-risk population is defined as people whose 10-year predicted risk are higher than the corresponding cut-off.

Multimedia Appendix 10-5: Performance of the esophageal cancer prediction model across different predicted risk cut-offs of full model in China Kadoorie Biobank using data-splitting.

| Cut-off (%) | Percent of high-risk population (%)^a^ | Sensitivity (%) | Specificity (%) | Youden's index (%) | Positive predictive value (%) | Negative predictive value (%) | Number needed to be screened to confirm one case | Missed cases per confirmed case |
| --- | --- | --- | --- | --- | --- | --- | --- | --- |
| 0.1 | 56.53 | 96.59 | 43.65 | 40.24 | 0.80 | 99.96 | 124.46 | 0.04 |
| 0.2 | 37.81 | 93.19 | 62.45 | 55.64 | 1.16 | 99.95 | 86.28 | 0.07 |
| 0.3 | 27.96 | 87.93 | 72.32 | 60.25 | 1.48 | 99.92 | 67.63 | 0.14 |
| 0.4 | 21.84 | 82.97 | 78.45 | 61.42 | 1.79 | 99.90 | 55.97 | 0.21 |
| 0.5 | 17.83 | 78.64 | 82.46 | 61.10 | 2.07 | 99.88 | 48.22 | 0.27 |
| 0.6 | 15.01 | 74.61 | 85.27 | 59.88 | 2.34 | 99.86 | 42.78 | 0.34 |
| 0.7 | 12.88 | 70.74 | 87.40 | 58.14 | 2.58 | 99.84 | 38.71 | 0.41 |
| 0.8 | 11.18 | 67.80 | 89.09 | 56.89 | 2.85 | 99.83 | 35.06 | 0.47 |
| 0.9 | 9.79 | 62.85 | 90.46 | 53.31 | 3.02 | 99.81 | 33.12 | 0.59 |
| 1.0 | 8.66 | 59.44 | 91.58 | 51.02 | 3.23 | 99.79 | 31.00 | 0.68 |
| 1.1 | 7.76 | 56.35 | 92.47 | 48.82 | 3.42 | 99.78 | 29.27 | 0.77 |
| 1.2 | 7.00 | 53.10 | 93.22 | 46.32 | 3.57 | 99.76 | 28.03 | 0.88 |
| 1.3 | 6.38 | 51.24 | 93.83 | 45.07 | 3.78 | 99.76 | 26.48 | 0.95 |
| 1.4 | 5.83 | 49.54 | 94.38 | 43.92 | 4.00 | 99.75 | 25.02 | 1.02 |
| 1.5 | 5.33 | 47.68 | 94.87 | 42.55 | 4.20 | 99.74 | 23.80 | 1.10 |
| 1.6 | 4.90 | 45.82 | 95.29 | 41.11 | 4.40 | 99.73 | 22.75 | 1.18 |
| 1.7 | 4.53 | 43.81 | 95.66 | 39.47 | 4.55 | 99.72 | 21.98 | 1.28 |
| 1.8 | 4.18 | 42.57 | 96.00 | 38.57 | 4.79 | 99.72 | 20.88 | 1.35 |
| 1.9 | 3.88 | 39.78 | 96.29 | 36.07 | 4.82 | 99.71 | 20.74 | 1.51 |
| 2.0 | 3.59 | 38.08 | 96.57 | 34.65 | 4.99 | 99.70 | 20.05 | 1.63 |

Full model was fitted to a random two-thirds of the China Kadoorie Biobank data and evaluated on the remaining one-third.

^a^High-risk population is defined as people whose 10-year predicted risk are higher than the corresponding cut-off.
