# Supplementary material for: Electronic Health Record–Based Absolute Risk Prediction Model for Esophageal Cancer in the Chinese Population: Model Development and External Validation
Source: JMIR Public Health Surveill. 2023 Mar 15;9:e43725. doi: 10.2196/43725 (PMC10132027; doi:10.2196/43725)
Supplement: Multimedia Appendix 11 [file publichealth_v9i1e43725_app11.docx]

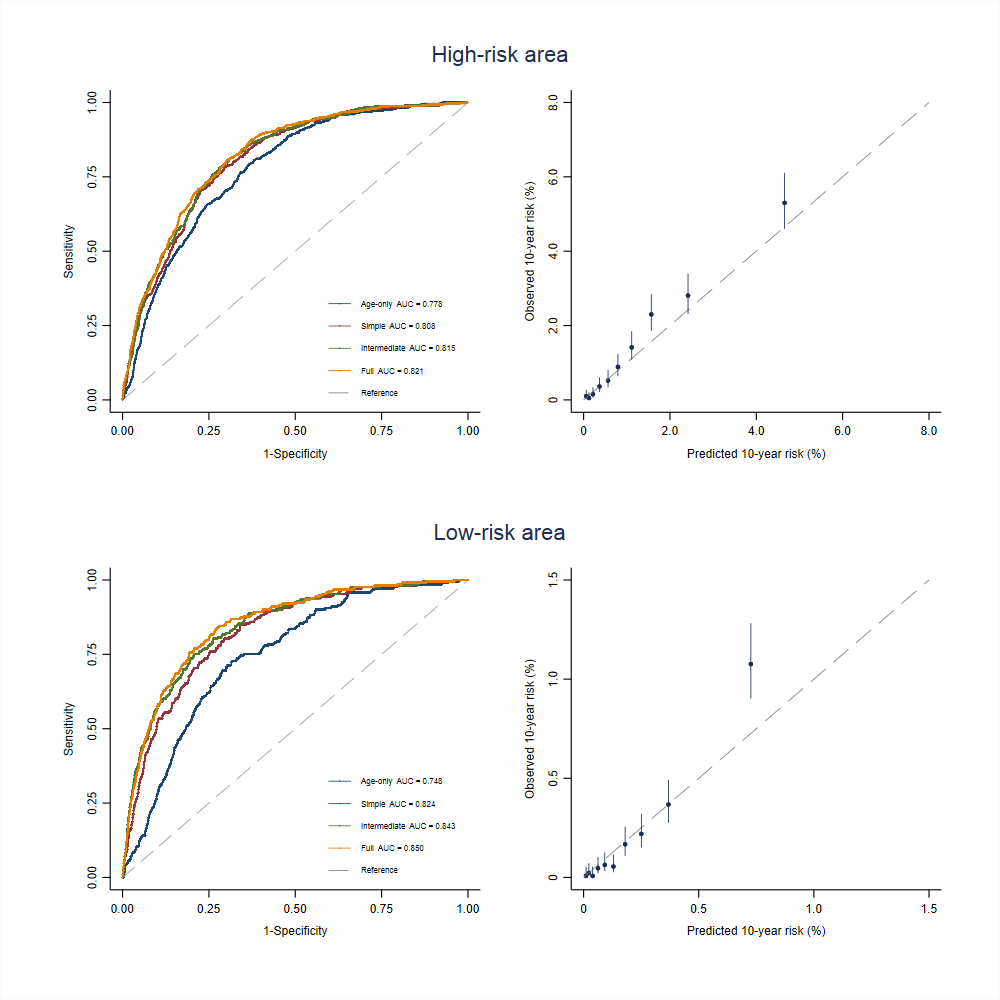


Multimedia Appendix 11-1: Discrimination and calibration of intermediate model in the high-risk (Hui county, Henan province and Pengzhou, Sichuan province) and low-risk (other study regions) area of China Kadoorie Biobank using data-splitting.

Receiver operating characteristic (ROC) curve and corresponding area under the ROC curve (AUC) (left); calibration plot (right).

Models were fitted to a random two-thirds of the China Kadoorie Biobank data and evaluated on the remaining one-third.

The observed 10-year risk was estimated by Kaplan-Meier analyses and plotted against model-predicted risk by decile.


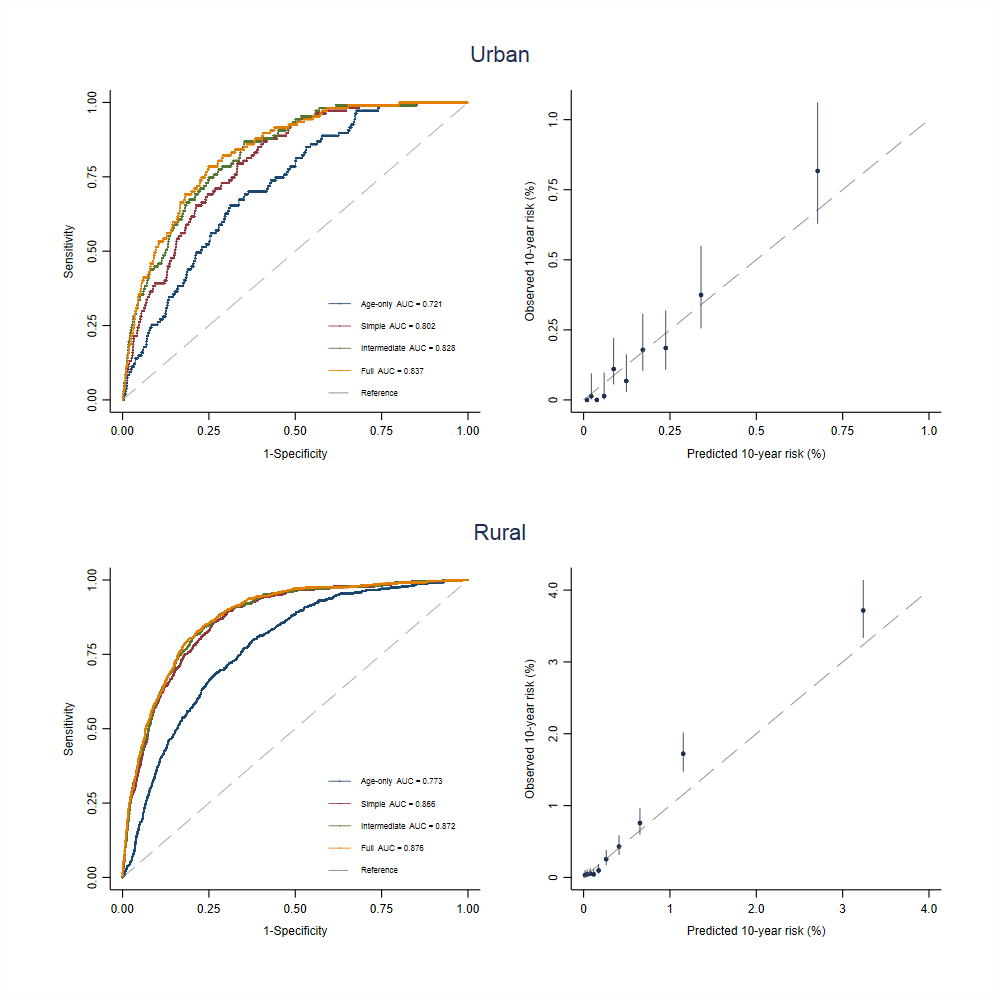


Multimedia Appendix 11-2: Discrimination and calibration of intermediate model in urban and rural areas of China Kadoorie Biobank using data-splitting.

Receiver operating characteristic (ROC) curve and corresponding area under the ROC curve (AUC) (left); calibration plot (right).

Models were fitted to a random two-thirds of the China Kadoorie Biobank data and evaluated on the remaining one-third.

The observed 10-year risk was estimated by Kaplan-Meier analyses and plotted against model-predicted risk by decile.


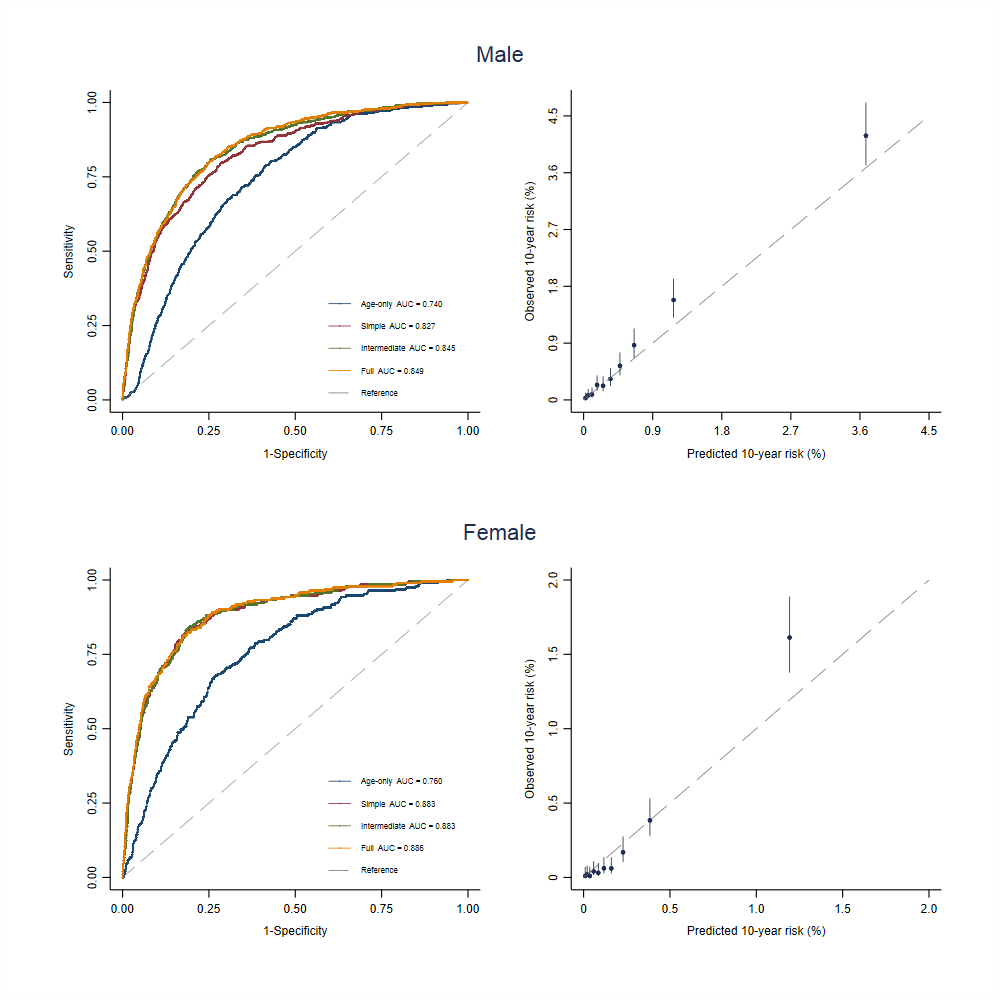


Multimedia Appendix 11-3: Discrimination and calibration of intermediate model among men and women of China Kadoorie Biobank using data-splitting.

Receiver operating characteristic (ROC) curve and corresponding area under the ROC curve (AUC) (left); calibration plot (right).

Models were fitted to a random two-thirds of the China Kadoorie Biobank data and evaluated on the remaining one-third.

The observed 10-year risk was estimated by Kaplan-Meier analyses and plotted against model-predicted risk by decile.


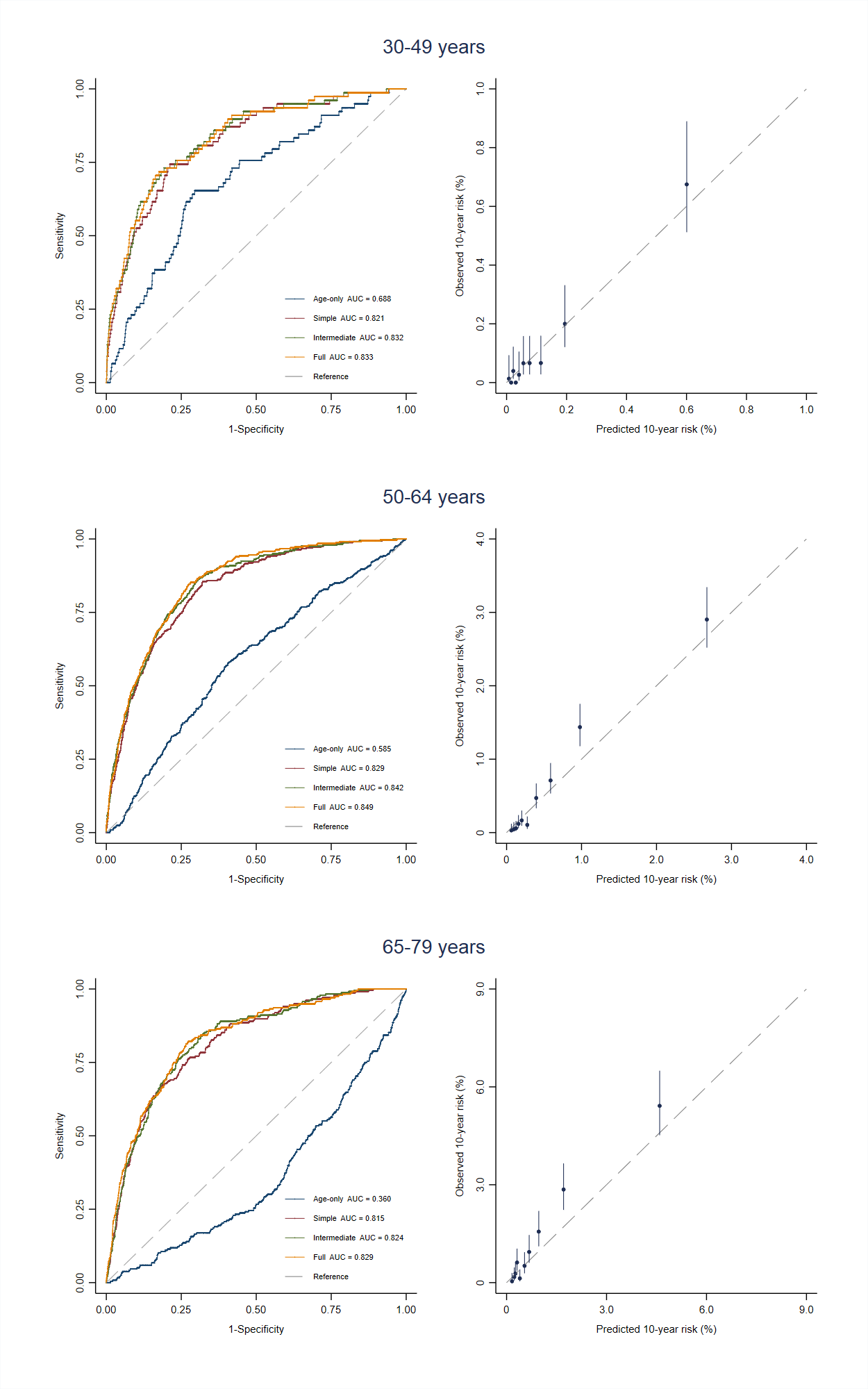


Multimedia Appendix 11-4: Discrimination and calibration of intermediate model by age groups in the China Kadoorie Biobank using data-splitting.

Receiver operating characteristic (ROC) curve and corresponding area under the ROC curve (AUC) (left); calibration plot (right).

Models were fitted to a random two-thirds of the China Kadoorie Biobank data and evaluated on the remaining one-third.

The observed 10-year risk was estimated by Kaplan-Meier analyses and plotted against model-predicted risk by decile.


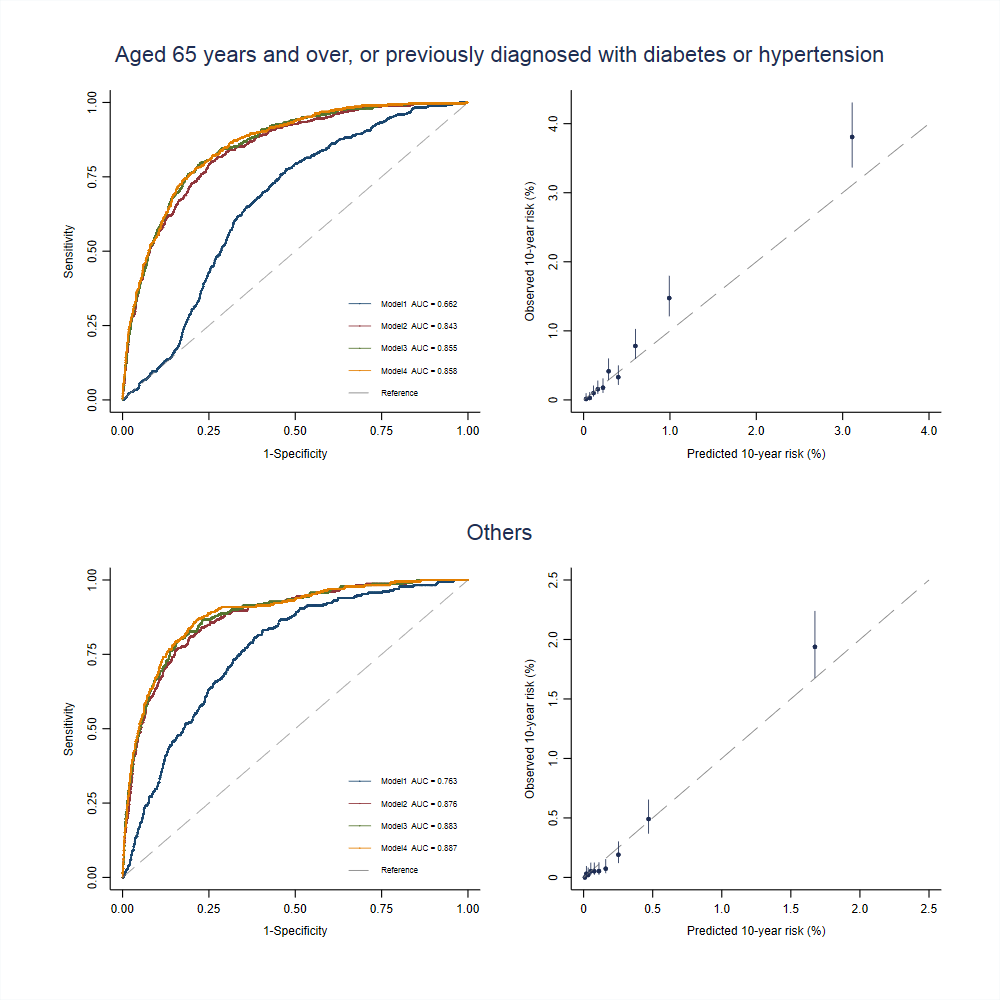
Multimedia Appendix 11-5: Discrimination and calibration of intermediate model among participants of China Kadoorie Biobank aged 65 years and older or with diabetes or hypertension at the baseline using data-splitting.

Receiver operating characteristic (ROC) curve and corresponding area under the ROC curve (AUC) (left); calibration plot (right).

Models were fitted to a random two-thirds of the China Kadoorie Biobank data and evaluated on the remaining one-third.

The observed 10-year risk was estimated by Kaplan-Meier analyses and plotted against model-predicted risk by decile.
