# Supplementary material for: Electronic Health Record–Based Absolute Risk Prediction Model for Esophageal Cancer in the Chinese Population: Model Development and External Validation
Source: JMIR Public Health Surveill. 2023 Mar 15;9:e43725. doi: 10.2196/43725 (PMC10132027; doi:10.2196/43725)
Supplement: Multimedia Appendix 12 [file publichealth_v9i1e43725_app12.docx]

Multimedia Appendix 12: Hazard ratios (95% CIs) for predictor variables of esophageal cancer prediction models developed separately in high-risk and low-risk^a^ area of the derivation subcohort of China Kadoorie Biobank.

|  | High-risk | | |  | Low-risk | | |
| --- | --- | --- | --- | --- | --- | --- | --- |
|  | Cases | Cases/PYs | Hazard ratio (95%CI) |  | Cases | Cases/PYs | Hazard ratio (95%CI) |
|  |  | (1/100,000) |  |  |  | (1/100,000) |  |
| Constant |  |  | 0.02 (0.01-0.03) |  |  |  | 0.00 (0.00-0.00) |
| Spline basis of age (knots:30, 60, 70, 90) |  |  |  |  |  |  |  |
| First |  |  | 3.41 (2.99-3.88) |  |  |  | 3.18 (2.66-3.79) |
| Second |  |  | 1.15 (1.06-1.24) |  |  |  | 1.13 (1.01-1.26) |
| Third |  |  | 1.00 (0.97-1.03) |  |  |  | 0.99 (0.95-1.03) |
| Sex |  |  |  |  |  |  |  |
| Male | 729 | 210.45 | Reference |  | 424 | 37.43 | Reference |
| Female | 385 | 76.00 | 0.42 (0.36-0.49) |  | 148 | 8.73 | 0.36 (0.28-0.46) |
| Highest education |  |  |  |  |  |  |  |
| Illiterate or primary school | 860 | 183.07 | Reference |  | 416 | 30.13 | Reference |
| Middle or high school | 248 | 67.01 | 0.71 (0.60-0.82) |  | 140 | 11.24 | 0.62 (0.50-0.76) |
| College or university | 6 | 45.92 | 0.47 (0.21-1.05) |  | 16 | 7.90 | 0.41 (0.24-0.68) |
| Family history of cancer |  |  |  |  |  |  |  |
| No | 729 | 107.64 | Reference |  |  |  |  |
| Yes | 385 | 219.10 | 2.01 (1.78-2.28) |  |  |  |  |
| Current smoking |  |  |  |  |  |  |  |
| No | 635 | 103.99 | Reference |  | 280 | 13.27 | Reference |
| Yes, cigarette or equivalent per day among smokers |  |  |  |  |  |  |  |
| <30 | 376 | 184.90 | 0.95 (0.82-1.11) |  | 251 | 40.53 | 1.22 (0.99-1.51) |
| ≥30 | 103 | 264.46 | 1.17 (0.93-1.49) |  | 41 | 41.13 | 1.28 (0.89-1.83) |
| Daily alcohol use |  |  |  |  |  |  |  |
| No | 901 | 118.23 | Reference |  | 404 | 15.59 | Reference |
| Yes, grams of pure alcohol per day among alcohol consumers |  |  |  |  |  |  |  |
| <30 | 15 | 124.75 | 0.68 (0.41-1.14) |  | 28 | 40.41 | 1.28 (0.87-1.89) |
| 30-59 | 53 | 217.74 | 1.09 (0.82-1.45) |  | 61 | 73.54 | 2.31 (1.74-3.06) |
| ≥60 | 145 | 265.91 | 1.52 (1.25-1.84) |  | 79 | 93.26 | 2.98 (2.30-3.87) |
| BMI, kg/m^2^ |  |  |  |  |  |  |  |
| <18.5 | 67 | 266.42 | Reference |  | 47 | 37.39 | Reference |
| 18.5-23.9 | 641 | 145.54 | 0.71 (0.55-0.91) |  | 338 | 22.96 | 0.85 (0.62-1.15) |
| ≥24.0 | 406 | 104.81 | 0.60 (0.46-0.78) |  | 187 | 15.19 | 0.68 (0.49-0.94) |
| Physical activity |  |  |  |  |  |  |  |
| Low | 889 | 134.97 | Reference |  |  |  |  |
| High^b^ | 225 | 115.81 | 0.80 (0.69-0.92) |  |  |  |  |
| Tea temperature preference |  |  |  |  |  |  |  |
| Non-daily a drinker or warm tea drinker |  |  |  |  | 388 | 16.81 | Reference |
| Hot tea |  |  |  |  | 107 | 28.24 | 0.98 (0.78-1.22) |
| Burning hot tea |  |  |  |  | 77 | 54.32 | 1.60 (1.24-2.06) |
| Fresh fruit consumption |  |  |  |  |  |  |  |
| Daily | 47 | 76.43 | Reference |  | 72 | 11.28 | Reference |
| Weekly | 225 | 74.29 | 0.79 (0.57-1.08) |  | 199 | 16.42 | 1.01 (0.76-1.33) |
| Less than weekly | 842 | 172.33 | 1.41 (1.05-1.90) |  | 301 | 30.74 | 1.41 (1.07-1.86) |

CI, confidence interval; PYs, person-years; BMI, body mass index.

^a^High-risk area denotes Hui county, Henan province and Pengzhou, Sichuan province in our study.

^b^High-level physical activity was defined as age- and sex-specific upper quarter of total physical activity level measured by metabolic equivalent of task-hours per day.
