# Supplementary material for: Electronic Health Record–Based Absolute Risk Prediction Model for Esophageal Cancer in the Chinese Population: Model Development and External Validation
Source: JMIR Public Health Surveill. 2023 Mar 15;9:e43725. doi: 10.2196/43725 (PMC10132027; doi:10.2196/43725)
Supplement: Multimedia Appendix 13 [file publichealth_v9i1e43725_app13.docx]

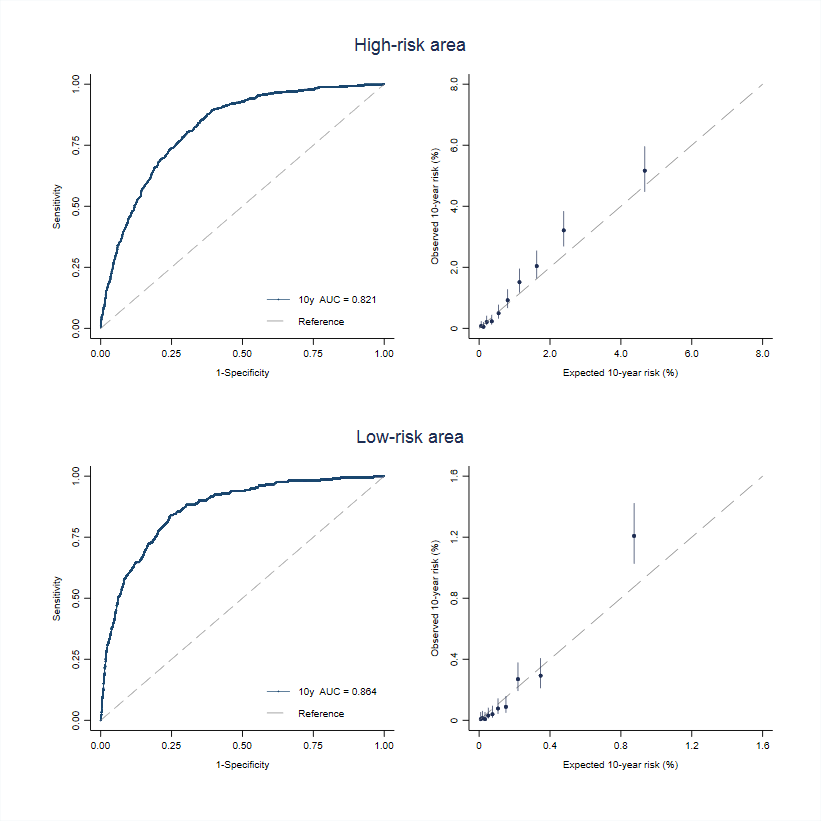


Multimedia Appendix 13: Model performance of two esophageal cancer prediction models developed separately in high-risk and low-risk areas of the derivation subcohort of China Kadoorie Biobank and applied in the corresponding validation subcohort.

Receiver operating characteristic (ROC) curve and corresponding area under the ROC curve (AUC) (left); calibration plot (right).

The observed 10-year risk was estimated by Kaplan-Meier and plotted against model-predicted risk by decile.
