# Supplementary material for: Electronic Health Record–Based Absolute Risk Prediction Model for Esophageal Cancer in the Chinese Population: Model Development and External Validation
Source: JMIR Public Health Surveill. 2023 Mar 15;9:e43725. doi: 10.2196/43725 (PMC10132027; doi:10.2196/43725)
Supplement: Multimedia Appendix 14 [file publichealth_v9i1e43725_app14.docx]

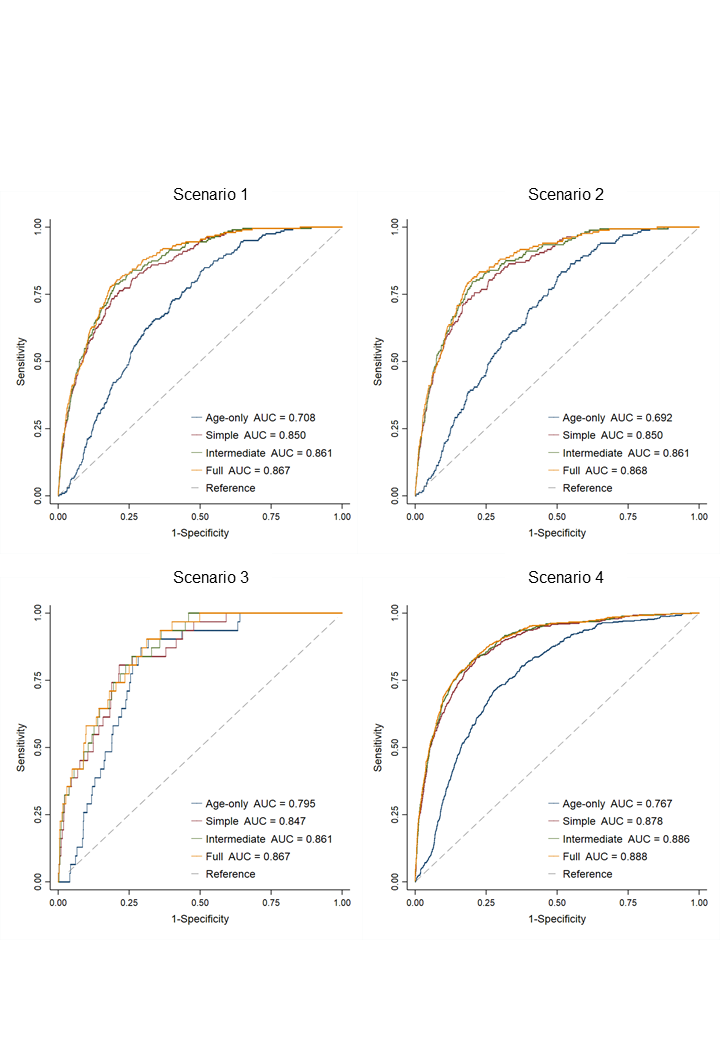


Multimedia Appendix 14: Discriminating ability of esophageal cancer prediction models in China Kadoorie Biobank using data-splitting in consideration of pathology reports.

AUC, area under the receiver operating characteristic curve.

Details of four scenarios are described in Multimedia Appendix 3. And models were fitted to a random two-thirds of the CKB data and evaluated on the remaining one-third.
