# Supplementary material for: Electronic Health Record–Based Absolute Risk Prediction Model for Esophageal Cancer in the Chinese Population: Model Development and External Validation
Source: JMIR Public Health Surveill. 2023 Mar 15;9:e43725. doi: 10.2196/43725 (PMC10132027; doi:10.2196/43725)
Supplement: Multimedia Appendix 15 [file publichealth_v9i1e43725_app15.docx]

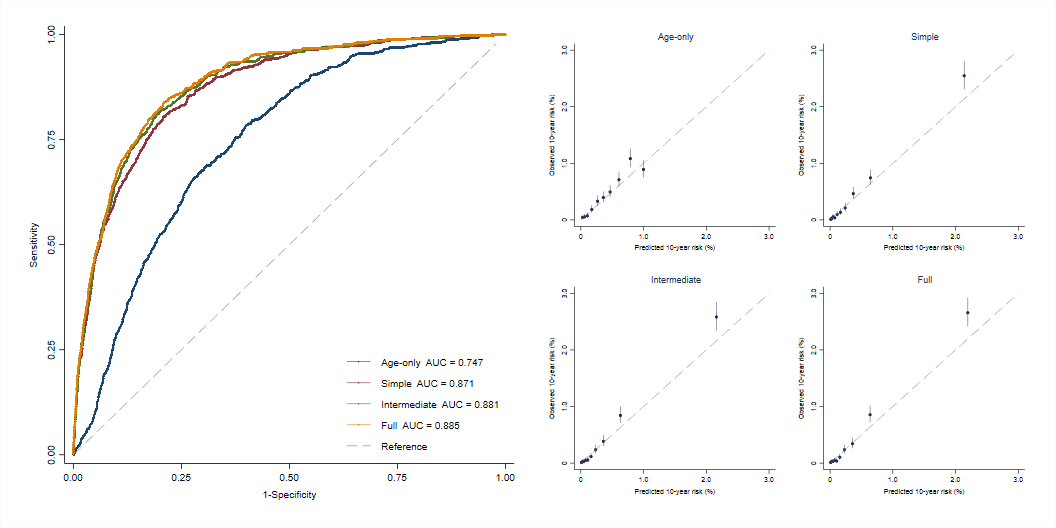
Multimedia Appendix 15: Model performance of esophageal cancer prediction models in China Kadoorie Biobank using data-splitting after excluding esophageal cancer cases occurring in the first year of follow-up.

Receiver operating characteristic (ROC) curve and corresponding area under the ROC curve (AUC) (left); calibration plot (right).

The observed 10-year risk was estimated by Kaplan-Meier analyses and plotted against model-predicted risk by decile.

A total of 228 esophageal cancer cases occurring in the first year of follow-up were excluded.

Models were fitted to a random two-thirds of the China Kadoorie Biobank data and evaluated on the remaining one-third.
