# Supplementary material for: Electronic Health Record–Based Absolute Risk Prediction Model for Esophageal Cancer in the Chinese Population: Model Development and External Validation
Source: JMIR Public Health Surveill. 2023 Mar 15;9:e43725. doi: 10.2196/43725 (PMC10132027; doi:10.2196/43725)
Supplement: Multimedia Appendix 16 [file publichealth_v9i1e43725_app16.zip › Instructions for user.docx]

**Introduction**

This repository provides Stata code to calculate the 10-year predicted risk of esophageal cancer for Chinese aged 30-79 years old based on supplied information and the four risk prediction models that were developed in China Kadoorie Biobank. Model 1- Model 4 refers to the age-only model, simple model, intermediate model, and full model. Details of the models see the main text.

The Stata code is done by modifying Dr. Muller’s code, which is available from Github, Inc. (https://github.com/dcmuller/ukbiobank_lca_model_predictions)

**Preparation**

- Stata, version 12 or higher
- User-written commands “stpm2” and “rcsgen” installed: “ssc install stpm2” and “ssc install rcsgen”
- Input_file: an ASCII plain text CSV file. The input file should contain the following variables. Details of the variables see the table below.

| **Model** | **Predictor** | **Description** | **Type** | **Category** | **Value** |
| --- | --- | --- | --- | --- | --- |
| Model 1 | age_at_study_date | Chronological age | Continuous |  | [30,79] |
| Model 2 | is_female | Sex | Categorical | Male | 0 |
|  |  |  |  | Female | 1 |
|  | high_risk_area | Living in areas with a high incidence of esophageal cancer | Categorical | No | 0 |
|  |  |  |  | Yes | 1 |
|  | region_is_urban | Living in urban areas | Categorical | Rural | 0 |
|  |  |  |  | Urban | 1 |
|  | education_3g | Education | Categorical | Illiterate or primary school | 0 |
|  |  |  |  | Middle or high school | 1 |
|  |  |  |  | College or university | 2 |
|  | cancer_fh_2g | Family history of cancer | Categorical | No | 0 |
|  |  |  |  | Yes | 1 |
| Model 3 | smoking_3g | Smoking status | Categorical | No | 1 |
|  |  |  |  | Yes, <30 cigarettes or equivalent per day | 2 |
|  |  |  |  | Yes, ≥30 cigarettes or equivalent per day | 3 |
|  | alcohol_4g | Alcohol drinking status | Categorical | No | 1 |
|  |  |  |  | Yes, <30 grams of pure alcohol per day | 2 |
|  |  |  |  | Yes, 30-59 grams of pure alcohol per day | 3 |
|  |  |  |  | Yes, ≥60 grams of pure alcohol per day | 4 |
|  | bmi_3g | Body mass index | Categorical | <18.5 | 1 |
|  |  |  |  | 18.5-23.9 | 2 |
|  |  |  |  | ≥24.0kg/m^2^ | 3 |
| Model 4 | PA_2g | Physical activity | Categorical | Not better than people of your age and sex | 0 |
|  |  |  |  | Better than people of your age and sex | 1 |
|  | hot_tea_3g | Hot tea preference | Categorical | Non-daily drinker or Warm | 1 |
|  |  |  |  | Hot | 2 |
|  |  |  |  | Burning hot | 3 |
|  | fruit_3g | Fresh fruit consumption | Categorical | Daily | 1 |
|  |  |  |  | Weekly | 2 |
|  |  |  |  | Less than weekly | 3 |

**Execution**

Open Stata and change working directory to the root of this repository. Running “**do predict_ec_10y_risk.do**” will calculate the 10-year absolute risk of esophageal cancer. By default, this will take predictor information from the file “input_file.csv”, calculate the absolute risk for each observation therein, and save the results in the file “output_file.csv”. The output file contains the same variables as the input file, with the addition of the variables “cif_inc10_model1”-“cif_inc10_model4”, which contain the predicted risk of our four models for each input observation, respectively.

Not that, if some observations have missing information on a certain predictor in the “input_file.csv”, then the predicted risk that required this predictor in the calculation will be missing. And if there is no column for a certain predictor, the predicted risk that required this predictor will be labeled as “NA”.

These default input, output, and time horizons can be changed by editing the parameters in the configuration block at the beginning of the file “predict_ec_10y_risk.do”.

**License**

The license below is also available from Github, Inc. (https://github.com/dcmuller/ukbiobank_lca_model_predictions)

-------------------------------------------------------------------------------------------------------

Copyright (c) 2015, dcmuller

All rights reserved.

Redistribution and use in source and binary forms, with or without modification, are permitted provided that the following conditions are met:

* Redistributions of source code must retain the above copyright notice, this list of conditions and the following disclaimer.

* Redistributions in binary form must reproduce the above copyright notice, this list of conditions and the following disclaimer in the documentation and/or other materials provided with the distribution.

* Neither the name of ukbiobank_lca_model_predictions nor the names of its contributors may be used to endorse or promote products derived from this software without specific prior written permission.

THIS SOFTWARE IS PROVIDED BY THE COPYRIGHT HOLDERS AND CONTRIBUTORS "AS IS"AND ANY EXPRESS OR IMPLIED WARRANTIES, INCLUDING, BUT NOT LIMITED TO, THE IMPLIED WARRANTIES OF MERCHANTABILITY AND FITNESS FOR A PARTICULAR PURPOSE ARE DISCLAIMED. IN NO EVENT SHALL THE COPYRIGHT HOLDER OR CONTRIBUTORS BE LIABLE FOR ANY DIRECT, INDIRECT, INCIDENTAL, SPECIAL, EXEMPLARY, OR CONSEQUENTIAL DAMAGES (INCLUDING, BUT NOT LIMITED TO, PROCUREMENT OF SUBSTITUTE GOODS OR SERVICES; LOSS OF USE, DATA, OR PROFITS; OR BUSINESS INTERRUPTION) HOWEVER CAUSED AND ON ANY THEORY OF LIABILITY, WHETHER IN CONTRACT, STRICT LIABILITY, OR TORT (INCLUDING NEGLIGENCE OR OTHERWISE) ARISING IN ANY WAY OUT OF THE USE OF THIS SOFTWARE, EVEN IF ADVISED OF THE POSSIBILITY OF SUCH DAMAGE.
